# USP21 functions as an oncogenic regulator of the Mdm2-p53 axis in colorectal cancer

**DOI:** 10.1038/s41420-026-03170-3

**Published:** 2026-05-22

**Authors:** Zhongyu Wang, Bo Yao, Weiran He, Xiaorui Guo, Ning Yu, Suyun Tang, Linzhu Luo, Fang Wang, Kailiang Zhao, Yide Mei

**Affiliations:** 1 Department of Thoracic Surgery, The First Affiliated Hospital of USTC, National Key Laboratory of Immune Response and Immunotherapy, Center for Advanced Interdisciplinary Science and Biomedicine of IHM, School of Basic Medical Sciences, Division of Life Sciences and Medicine, University of Science and Technology of China, Hefei, China; 2 Zhejiang University-University of Edinburgh Institute, Zhejiang University, Haining, China; 3 School of Basic Medical Sciences, Anhui Medical University, Hefei, China

**Keywords:** Cell signalling, Oncogenes

## Abstract

The tumor suppressor p53 is a pivotal guardian against tumorigenesis, with its activity primarily constrained by the ubiquitin E3 ligase Mdm2. However, the full complexity of the Mdm2-p53 regulatory network remains elusive. Here we report that the deubiquitinating enzyme USP21 physically interacts with and stabilizes Mdm2 in a deubiquitinase activity-independent manner. Mechanistically, USP21 acts as a scaffold to facilitate the USP7-Mdm2 interaction, enhancing Mdm2 stability and consequently promoting p53 ubiquitination and degradation. Functionally, USP21-mediated p53 suppression attenuates its tumor suppressive activity and accelerates colorectal cancer progression. Clinically, USP21 is upregulated in colorectal cancer tissues, and its elevated expression correlates with poor overall survival in patients with wild-type p53 tumors, but not in those with p53 mutations. These findings establish USP21 as an important regulator of the Mdm2-p53 axis and reveal its critical role in promoting colorectal carcinogenesis via p53 inhibition.

## Introduction

The tumor suppressor p53 plays a prominent role in preventing tumorigenesis by orchestrating cellular stress responses that trigger anti-proliferative mechanisms, including cell cycle arrest, apoptosis, and senescence [1, 2]. Beyond its canonical functions, p53 also regulates diverse cellular processes such as metabolic adaptation, autophagy, and ferroptosis [3]. In human cancers, p53 is frequently inactivated through mutations or deletions, with over half of tumors exhibiting loss of p53 function [4]. Even in tumors retaining wild-type p53, the pathway is often compromised due to aberrant signaling in upstream or downstream components, facilitating tumor progression [5, 6].

The anti-proliferative activity of p53 is tightly regulated to prevent its inappropriate activation under non-stress conditions. This control is primarily mediated by Mdm2, a ubiquitin E3 ligase that promotes p53 ubiquitination and proteasomal degradation [7–9]. Mdm2 not only targets p53 for degradation via its trans-E3 ligase activity but also autoregulates its own stability through self-ubiquitination, ensuring its rapid turnover [10, 11]. The critical role of Mdm2 in p53 regulation is underscored by the embryonic lethality of Mdm2-null mice, a phenotype entirely rescued by concurrent p53 deletion [12, 13]. Under stress conditions, Mdm2-mediated p53 degradation is inhibited, enabling p53 accumulation and activation of its tumor-suppressive functions [14]. Given its central role in restraining p53, Mdm2 itself acts as an oncogene, with its amplification or overexpression commonly observed in cancers harboring wild-type p53 [15]. Therefore, elucidating the regulatory mechanisms governing Mdm2 is essential for advancing our understanding of p53 biology and its implication in cancer development.

Ubiquitin-specific protease 7 (USP7), also known as herpesvirus-associated ubiquitin-specific protease (HAUSP), is a critical regulator of the Mdm2-p53 signaling axis [16, 17]. USP7 exhibits a dual role in p53 regulation: it can directly stabilize p53 through deubiquitination or indirectly promote p53 degradation by increasing Mdm2 stability [18–20]. Under normal conditions, USP7 preferentially binds to Mdm2, facilitating p53 degradation. However, in response to DNA damage or other stress signals, ATM-mediated phosphorylation disrupts the USP7-Mdm2 interaction, shifting USP7’s binding preference toward p53 and leading to its stabilization [21–23]. This dynamic and context-dependent regulation of both Mdm2 and p53 by USP7 highlights its pivotal yet complex role in maintaining cellular homeostasis and emphasizes the necessity of identifying novel USP7 modulators.

Ubiquitin-specific protease 21 (USP21) is a member of the ubiquitin-specific protease (USP) family, a class of deubiquitinating enzymes. Structurally, USP21 consists of an unstructured N-terminal region and a C-terminal catalytic USP domain. USP21 plays an important role in regulating multiple cancer-associated signaling pathways, including the Hippo, NF-κB, MAPK, Wnt/β-catenin, ERK, EGFR, and KRAS signaling [24–30]. USP21 also modulates cell cycle progression, Treg lineage stability, and stemness maintenance in mouse embryonic stem cells [31–34]. Its primary mechanism involves deubiquitination of key proteins within these pathways. However, the non-enzymatic functions of USP21 remain poorly understood.

In this study, we identify USP21 as a negative regulator of p53 that promotes colorectal cancer progression. Mechanistically, USP21 strengthens the USP7-Mdm2 interaction and stabilizes Mdm2 independently of its deubiquitinase activity, ultimately enhancing Mdm2-mediated ubiquitination and degradation of p53. Functionally, USP21 negatively regulates the tumor suppressive activity of p53, thereby accelerating colorectal cancer progression. Clinically, USP21 is overexpressed in colorectal cancer tissues, and high USP21 expression is associated with poor overall survival in patients with wild-type p53 colorectal cancer, but not in those with p53-mutated colorectal cancer. Collectively, these findings highlight USP21’s critical role in the Mdm2-p53 signaling pathway and suggest its potential as a therapeutic target for colorectal cancer.

## Results

### USP21 is an Mdm2-interacting protein

**Fig. 1:**
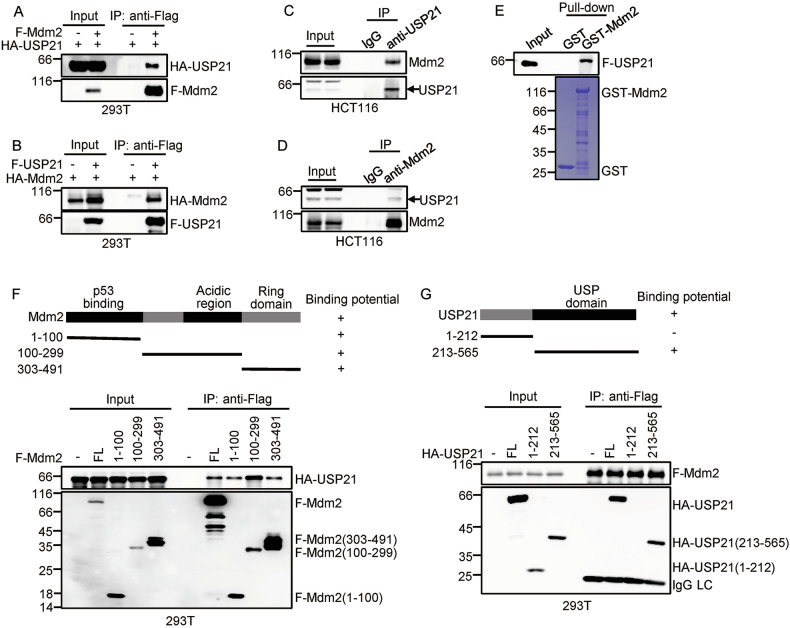
USP21 interacts with Mdm2. **A** Lysates from HEK293T cells expressing either HA-USP21 alone or together with Flag-Mdm2 were subjected to immunoprecipitation analysis. **B** Lysates from HEK293T cells expressing either HA-Mdm2 alone or together with Flag-USP21 were subjected to immunoprecipitation analysis. **C** Lysates from HCT116 cells were immunoprecipitated with anti-USP21 antibody or an isotype-matched control IgG. **D** Lysates from HCT116 cells were immunoprecipitated with anti-Mdm2 antibody or an isotype-matched control IgG. **E** Recombinant GST or GST-Mdm2 proteins immobilized on glutathione beads were incubated with purified Flag-USP21. Both input and bead-bound proteins were analyzed by western blotting. **F** Lysates from HEK293T cells expressing either HA-USP21 alone or together with the indicated Flag-tagged Mdm2 mutants were subjected to immunoprecipitation analysis. **G** Lysates from HEK293T cells expressing either Flag-Mdm2 alone or together with the indicated HA-tagged USP21 mutants were subjected to immunoprecipitation analysis.

Given the pivotal role of the Mdm2-p53 signaling axis in tumorigenesis, we sought to elucidate novel regulatory mechanisms governing this pathway by identifying previously unrecognized Mdm2-interacting proteins. To this end, we performed mass spectrometry-based proteomic analysis of anti-Mdm2 immunoprecipitates from HCT116 cells harboring wild-type p53. This approach led to the identification of USP21 as a potential Mdm2-binding partner (Supplementary Table 1). To confirm this interaction, we conducted co-immunoprecipitation assays in HEK293T cells overexpressing tagged proteins, which demonstrated a specific interaction between exogenously expressed USP21 and Mdm2 (Fig. 1A, B). We also validated this interaction at the endogenous level though reciprocal co-immunoprecipitation assays using anti-USP21 and anti-Mdm2 antibodies (Fig. 1C, D). Moreover, an in vitro GST pull-down assay revealed that USP21 directly associated with Mdm2 (Fig. 1E).

To define the regions mediating the interaction between USP21 and Mdm2, we generated a series of deletion mutants for both proteins (Fig. 1F, G). Immunoprecipitation analysis revealed that, similar to full-length Mdm2, all three tested Mdm2 deletion mutants interacted with USP21 (Fig. 1F), implying that Mdm2 likely binds to USP21 through multiple regions. For USP21, the C-terminal USP domain (aa 213–565) of USP21 was able to interact with Mdm2 (Fig. 1G). In contrast, the N-terminal region (aa 1–202) of USP21 failed to associate with Mdm2 (Fig. 1G), suggesting that the USP domain of USP21 mediates the interaction with Mdm2. Collectively, these findings establish USP21 as a novel binding partner of Mdm2.

### USP21 enhances Mdm2 protein stability

**Fig. 2:**
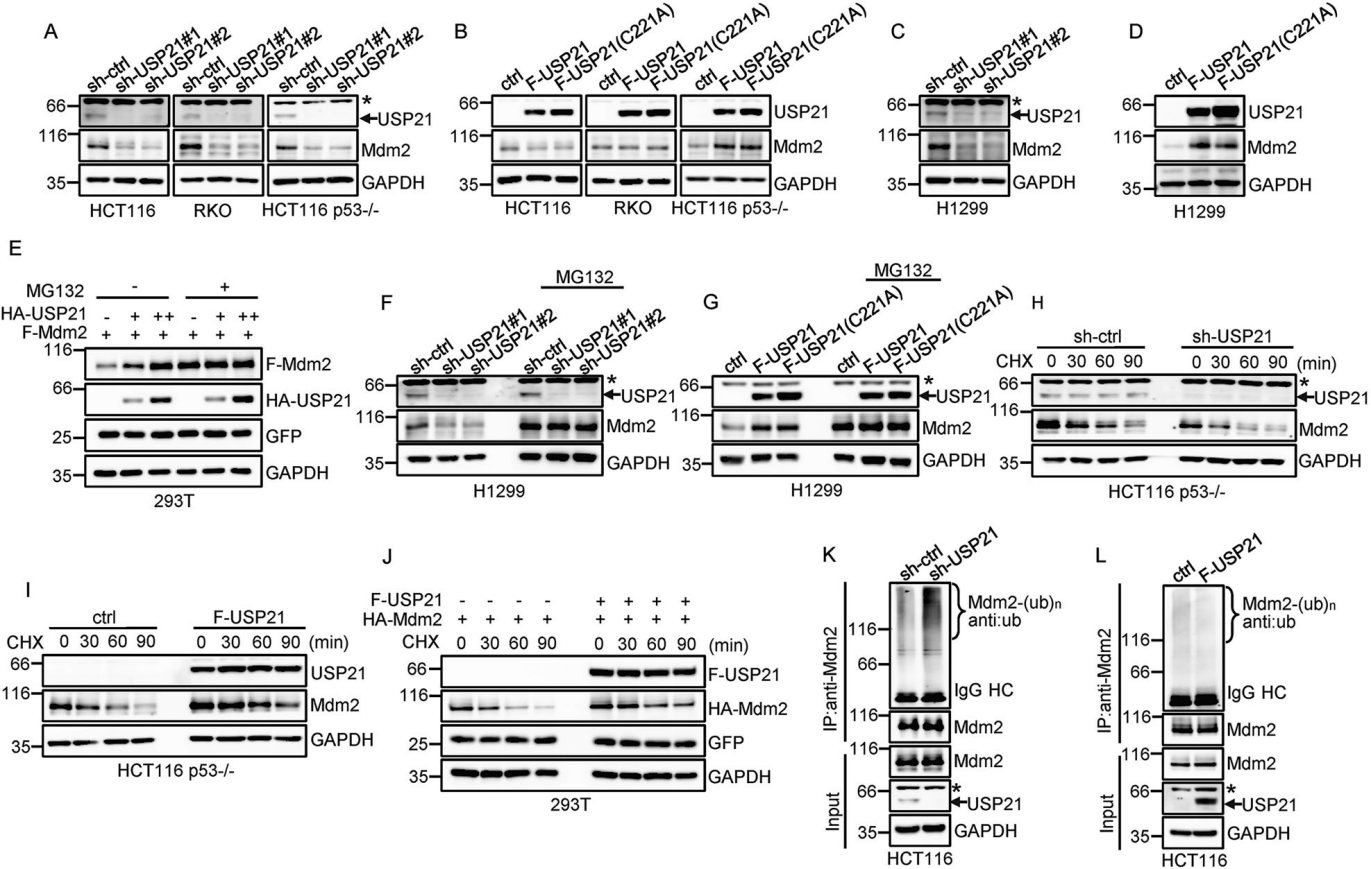
USP21 stabilizes Mdm2. **A** Western blot analysis of lysates from the indicated cells transduced with lentiviruses expressing control shRNA, USP21 shRNA#1, or USP21 shRNA#2. * indicates a non-specific band. **B** Western blot analysis of lysates from the indicated cells transduced with lentiviruses expressing control, Flag-USP21, or Flag-USP21(C221A). **C** Western blot analysis of lysates from H1299 cells transduced with lentiviruses expressing control shRNA, USP21 shRNA#1, or USP21 shRNA#2. * indicates a non-specific band. **D** Western blot analysis of lysates from H1299 cells transduced with lentiviruses expressing control, Flag-USP21, or Flag-USP21(C221A). **E** HEK293T cells were transfected with either Flag-Mdm2 alone or together with increasing amounts of HA-USP21. Twenty-four hours later, cells were treated with or without 10 μM MG132 for an additional 2 h, followed by western blot analysis. SE and LE indicate short-time and long-time exposure, respectively. Levels of GFP and GAPDH were used as controls for transfection efficiency and sample loading, respectively. **F** H1299 cells transduced with lentiviruses expressing control shRNA, USP21 shRNA#1, or USP21 shRNA#2 were treated with or without 20 μM MG132 for 6 h, followed by western blot analysis. * indicates a non-specific band. **G** H1299 cells transduced with lentiviruses expressing control, Flag-USP21, or Flag-USP21(C221A) were treated with or without 20 μM MG132 for 6 h, followed by western blot analysis. * indicates a non-specific band. **H** p53^–/–^ HCT116 cells transduced with lentiviruses expressing control shRNA or USP21 shRNA were treated with 20 μg/mL cycloheximide (CHX) for the indicated periods of time, followed by western blot analysis. * indicates a non-specific band. **I** p53^–/–^ HCT116 cells transduced with lentiviruses expressing control or Flag-USP21 were treated with 20 μg/mL cycloheximide (CHX) for the indicated periods of time, followed by western blot analysis. **J** HEK293T cells overexpressing HA-Mdm2 alone or HA-Mdm2 plus Flag-USP21 were treated with 20 μg/mL cycloheximide (CHX) for the indicated periods of time, followed by western blot analysis. **K** HCT116 cells transduced with lentiviruses expressing control shRNA or USP21 shRNA were treated with 20 μM MG132 for 6 h, followed by an in vivo ubiquitination assay. * indicates a non-specific band. **L** HCT116 cells transduced with lentiviruses expressing control or Flag-USP21 were treated with 20 μM MG132 for 6 h, followed by an in vivo ubiquitination assay. * indicates a non-specific band.

Given that USP21 directly interacts with Mdm2, we next investigated whether USP21 regulates Mdm2 levels. In both wild-type p53 (HCT116 and RKO) and p53-deficient (p53^-/-^ HCT116) cells, knockdown of USP21 greatly reduced Mdm2 levels (Fig. 2A). Surprisingly, ectopic expression of USP21 in HCT116 and RKO cells failed to increase Mdm2 levels (Fig. 2B). We hypothesized that the inability of USP21 to elevate Mdm2 in wild-type p53 cells might be due to the presence of a self-regulatory loop between p53 and Mdm2 [35, 36]. Consistent with this idea, USP21 overexpression markedly elevated Mdm2 levels in p53^-/-^ HCT116 cells (Fig. 2B). Moreover, in p53-null H1299 cells, USP21 knockdown decreased Mdm2 levels, while its overexpression increased Mdm2 levels (Fig. 2C, D), further supporting the positive effect of USP21 on Mdm2 expression. Notably, the ability of USP21 to enhance Mdm2 expression appeared to be independent of its deubiquitinase activity, as the catalytically inactive USP21 (C221A) mutant still increased Mdm2 levels in p53^-/-^ HCT116 and H1299 cells (Fig. 2B, D). Furthermore, the USP21 fragment (aa 213–565) containing the Mdm2-binding USP domain also upregulated Mdm2, whereas the Mdm2 binding-defective mutant of USP21 (aa 1–212) had no such effect (Fig. S1A). Collectively, these data indicate that the interaction with Mdm2 is essential for USP21 to enhance Mdm2 levels.

To determine how USP21 enhances Mdm2 levels, we evaluated its effect on Mdm2 stability. In HEK293T cells, co-expression of USP21 increased Mdm2 levels in a dose-dependent manner (Fig. 2E). However, this effect was abolished upon treatment with the proteasome inhibitor MG132 (Fig. 2E). In addition, in H1299 cells, USP21 knockdown reduced Mdm2 levels, whereas overexpression of either wild-type USP21 or USP21(C221A) elevated Mdm2 levels in the absence, but not presence of MG132 (Fig. 2F, G), suggesting that USP21 prevents Mdm2 from proteasomal degradation. The subsequent cycloheximide chase assays in p53^-/-^ HCT116 cells showed that USP21 knockdown accelerated Mdm2 degradation, whereas USP21 overexpression extended Mdm2’s half-life (Fig. 2H, I and S1B, C). Co-expression experiments in HEK293T cells also confirmed the stabilizing effect of USP21 on Mdm2 (Fig. 2J and S1D). Mechanistically, ubiquitination assays showed that USP21 negatively regulated Mdm2 ubiquitination. Knockdown of USP21 enhanced, whereas USP21 overexpression reduced, ubiquitination of endogenous Mdm2 (Fig. 2K, L). Taken together, these data suggest that USP21 stabilizes Mdm2 by inhibiting its ubiquitin-dependent proteasomal degradation.

### USP21 coordinates with USP7 to stabilize Mdm2

**Fig. 3:**
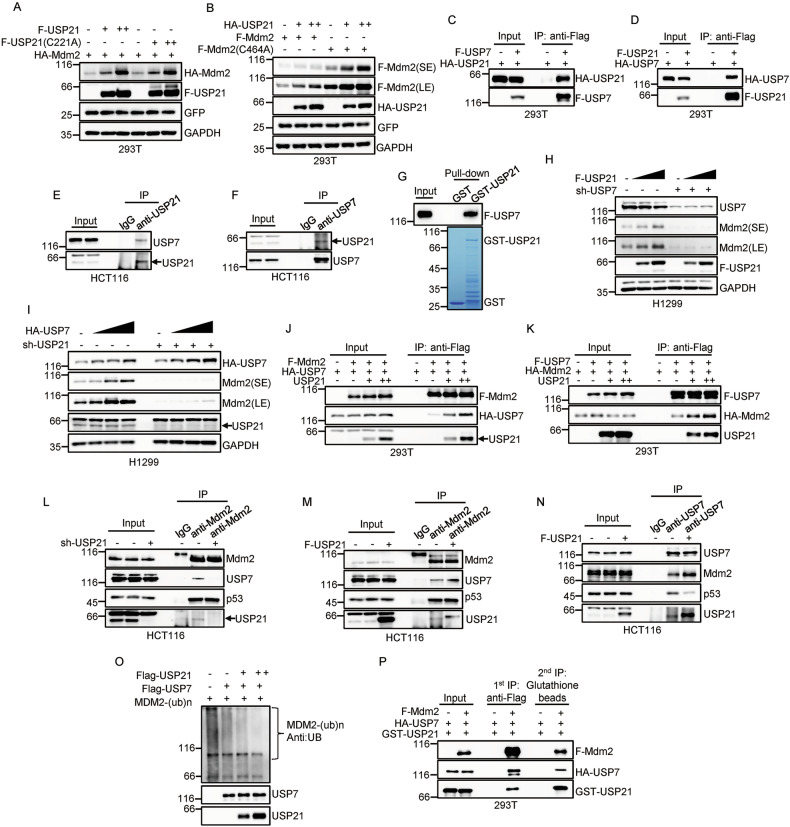
USP21 coordinates with USP7 to stabilize Mdm2. **A** HEK293T cells were transfected with either HA-Mdm2 alone or together with increasing amounts of the indicated Flag-USP21 constructs. Forty-eight hours later, cell lysates were analyzed by western blotting. **B** HEK293T cells were transfected with either Flag-Mdm2 alone, Flag-Mdm2(C464A) alone, or in combination with increasing amounts of HA-USP21. Forty-eight hours later, cell lysates were analyzed by western blotting. **C** Lysates from HEK293T cells expressing either HA-USP21 alone or together with Flag-USP7 were subjected to immunoprecipitation analysis. **D** Lysates from HEK293T cells expressing either HA-USP7 alone or together with Flag-USP21 were subjected to immunoprecipitation analysis. **E** Lysates from HCT116 cells were immunoprecipitated with anti-USP21 antibody or an isotype-matched control IgG. **F** Lysates from HCT116 cells were immunoprecipitated with anti-USP7 antibody or an isotype-matched control IgG. **G** Recombinant GST or GST-USP21 proteins immobilized on glutathione beads were incubated with purified Flag-USP7. Both input and bead-bound proteins were analyzed by western blotting. **H** The control or USP7 knockdown H1299 cells were infected with lentiviruses expressing control or Flag-USP21 as indicated. Forty-eight hours later, cell lysates were analyzed by western blotting. **I** The control or USP21 knockdown H1299 cells were transfected with increasing amounts of HA-USP7. Twenty-four hours later, cell lysates were analyzed by western blotting. **J** HEK293T cells were transfected with Flag-Mdm2, HA-USP7, and USP21 in the indicated combinations. Twenty-four hours later, cells were treated with 20 μM MG132 for an additional 6 h, followed by immunoprecipitation analysis. **K** HEK293T cells were transfected with Flag-USP7, HA-Mdm2, and USP21 in the indicated combinations. Twenty-four hours later, cells were treated with 20 μM MG132 for an additional 6 h, followed by immunoprecipitation analysis. **L** HCT116 cells transduced with lentiviruses expressing control shRNA or USP21 shRNA were treated with 20 μM MG132 for 6 h, followed by immunoprecipitation analysis. **M** HCT116 cells transduced with lentiviruses expressing control or Flag-USP21 were treated with 20 μM MG132 for 6 h, followed by immunoprecipitation analysis with anti-Mdm2 antibody. **N** HCT116 cells transduced with lentiviruses expressing control or Flag-USP21 were treated with 20 μM MG132 for 6 h, followed by immunoprecipitation analysis with anti-USP7 antibody. **O** Ubiquitinated Flag-Mdm2 purified from HEK293T cells was incubated with purified FLAG-USP7 and FLAG-USP21 as indicated. The reaction mixtures were analyzed by western blotting. **P** HA-USP7 and GST-USP21 were co-transfected into HEK293T cells in the presence or absence of Flag-Mdm2. Twenty-four hours later, cell lysates were first immunoprecipitated with anti-Flag antibody. Flag-Mdm2 and its interacting proteins were eluted with 3×Flag peptide. One half of the eluent was analyzed by western blotting, while the other half was subjected to a second immunoprecipitation with glutathione beads.

We next explored how USP21 enhances Mdm2 stability. Similar to wild-type USP21, the catalytically inactive USP21 (C221) mutant was able to dose-dependently increase Mdm2 levels upon overexpression (Fig. 3A), further confirming that USP21 stabilizes Mdm2 through a mechanism independent of its deubiquitinase activity. Interestingly, when co-expressed with USP21, the ubiquitin E3 ligase-inactive Mdm2 mutant (C464A), devoid of autoubiquitination, was still upregulated (Fig. 3B). This suggests that USP21-mediated Mdm2 stabilization does not result from the inhibition of Mdm2 autoubiquitination and degradation.

USP7, a well-established deubiquitinating enzyme, is known to stabilize Mdm2 under non-stress conditions [19]. To determine whether USP7 is involved in USP21-mediated Mdm2 stabilization, we first examined their interaction. Co-immunoprecipitation assays revealed a strong association between exogenously expressed USP21 and USP7 (Fig. 3C, D). This interaction was also verified at the endogenous level using reciprocal co-immunoprecipitation assays with anti-USP21 and anti-USP7 antibodies (Fig. 3E, F). An in vitro GST pull-down assay with purified GST-USP21 and Flag-USP7 further demonstrated a direct binding between these two proteins (Fig. 3G). To map the interaction regions, we generated a series of deletion mutants for both USP21 and USP7 (Fig. S2A, B). Truncation analysis showed that USP7 interacted with USP21 through multiple regions outside its catalytic domain (aa 208–506), while the USP domain of USP21 (aa 213–526) was responsible for its binding to USP7 (Fig. S2A, B). Since the USP domain of USP21 (aa 213–526) mediates the interaction with both Mdm2 and USP7 (Fig. 1G and S2B), we subdivided it into two regions: USP21 (aa 213–390) and USP21 (aa 391–565). Immunoprecipitation assays showed that USP7 bound to both fragments, while Mdm2 selectively interacted with USP21 (aa 391–565) (Fig. S2C, D).

To further investigate whether USP21-induced Mdm2 stabilization depends on USP7, we performed rescue experiments in p53-null H1299 cells. As expected, USP21 increased endogenous Mdm2 levels in control H1299 cells (Fig. 3H). However, this effect was significantly diminished when USP7 was knocked down (Fig. 3H). In addition, when USP21 was silenced in H1299 cells, the ability of USP7 to elevate Mdm2 levels was also attenuated (Fig. 3I), indicating mutual dependence between USP21 and USP7 in stabilizing Mdm2. We next examined whether USP21 modulates the USP7-Mdm2 interaction. Under overexpression conditions, USP21 was shown to dose-dependently enhance the interaction between USP7 and Mdm2 (Fig. 3J, K). Furthermore, knockdown of USP21 diminished the endogenous USP7-Mdm2 binding, while USP21 overexpression strengthened it (Fig. 3L, M). Given that USP7 interacts with and stabilizes both Mdm2 and p53, we proposed that USP21 could modulate this balance by promoting USP7’s association with Mdm2 over p53. Subsequent co-immunoprecipitation assays validated that USP21 overexpression increased USP7-Mdm2 binding and concurrently decreased USP7-p53 binding (Fig. 3N). Consistent with this shift, USP21 was shown to enhance USP7-catalyzed deubiquitination of Mdm2 (Fig. 3O). By performing sequential immunoprecipitation experiments, we showed that USP21, USP7, and Mdm2 formed a ternary complex (Fig. 3P), indicating that USP21 acts as a scaffold to facilitate the interaction between USP7 and Mdm2, thereby promoting USP7-mediated deubiquitination and stabilization of Mdm2. Together, these data suggest that USP21 cooperates with USP7 to stabilize Mdm2.

### USP21 facilitates Mdm2-dependent ubiquitination and degradation of p53

**Fig. 4:**
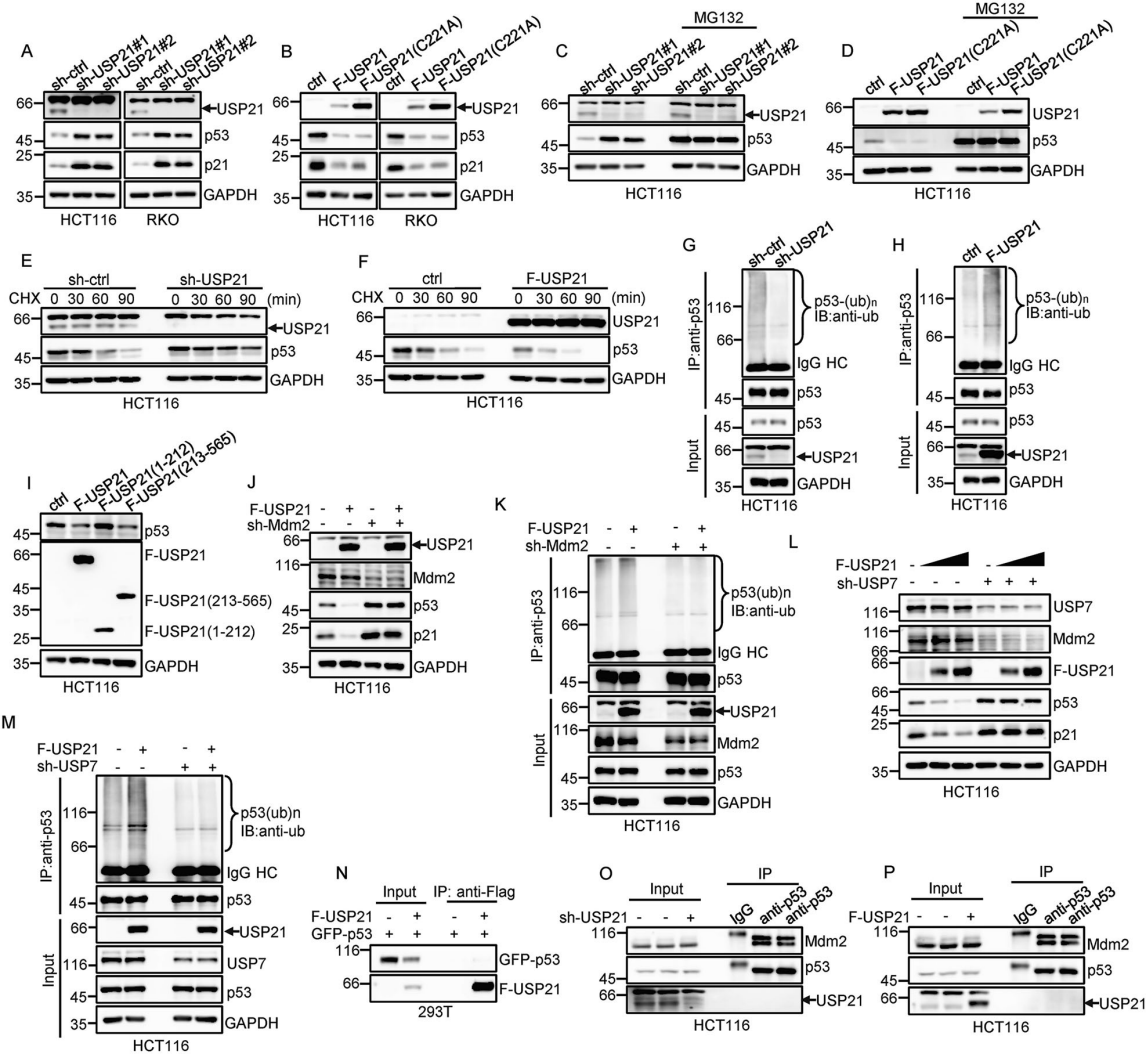
USP21 promotes Mdm2-dependent p53 ubiquitination and degradation. **A** Western blot analysis of lysates from HCT116 and RKO cells transduced with lentiviruses expressing control shRNA, USP21 shRNA#1, or USP21 shRNA#2. **B** Western blot analysis of lysates from HCT116 and RKO cells transduced with lentiviruses expressing control, Flag-USP21, or Flag-USP21(C221A). **C** HCT116 cells transduced with lentiviruses expressing control shRNA, USP21 shRNA#1, or USP21 shRNA#2 were treated with or without 20 μM MG132 for 6 h, followed by western blot analysis. **D** HCT116 cells transduced with lentiviruses expressing control, Flag-USP21, or Flag-USP21(C221A) were treated with or without 20 μM MG132 for 6 h, followed by western blot analysis. **E** HCT116 cells transduced with lentiviruses expressing control shRNA or USP21 shRNA were treated with 20 μg/mL cycloheximide (CHX) for the indicated periods of time, followed by western blot analysis. **F** HCT116 cells transduced with lentiviruses expressing control or Flag-USP21 were treated with 20 μg/mL cycloheximide (CHX) for the indicated periods of time, followed by western blot analysis. **G** HCT116 cells transduced with lentiviruses expressing control shRNA or USP21 shRNA were treated with 20 μM MG132 for 6 h, followed by an in vivo ubiquitination assay. **H** HCT116 cells transduced with lentiviruses expressing control or Flag-USP21 were treated with 20 μM MG132 for 6 h, followed by an in vivo ubiquitination assay. **I** Western blot analysis of lysates from HCT116 cells transduced with lentiviruses expressing control, Flag-USP21, Flag-USP21(1–212), or Flag-USP21(213–565). **J** Western blot analysis of lysates from HCT116 cells transduced with lentiviruses expressing control, Flag-USP21, Mdm2 shRNA, or Flag-USP21 plus Mdm2 shRNA. **K** HCT116 cells transduced with lentiviruses expressing control, Flag-USP21, Mdm2 shRNA, or Flag-USP21 plus Mdm2 shRNA were treated with 20 μM MG132 for 6 h, followed by an in vivo ubiquitination assay. **L** The control or USP7 knockdown HCT116 cells were infected with lentiviruses expressing control or Flag-USP21 as indicated. Forty-eight hours later, cell lysates were analyzed by western blotting. **M** HCT116 cells transduced with lentiviruses expressing control, Flag-USP21, USP7 shRNA, or Flag-USP21 plus USP7 shRNA were treated with 20 μM MG132 for 6 h, followed by an in vivo ubiquitination assay. **N** Lysates from HEK293T cells expressing either GFP-p53 alone or together with Flag-USP21 were subjected to immunoprecipitation analysis. **O** HCT116 cells transduced with lentiviruses expressing control shRNA or USP21 shRNA were treated with 20 μM MG132 for 6 h, followed by immunoprecipitation analysis. **P** HCT116 cells transduced with lentiviruses expressing control or Flag-USP21 were treated with 20 μM MG132 for 6 h, followed by immunoprecipitation analysis.

The stabilizing effect of USP21 on Mdm2 prompted us to ask whether USP21 regulates p53 through Mdm2. We first evaluated the effect on USP21 on p53 levels. Knockdown of USP21 increased, whereas ectopic expression of USP21 decreased, the protein levels of both p53 and its downstream target p21 in p53 wild-type cancer cell lines (HCT116 and RKO) (Fig. 4A, B). However, neither knockdown nor overexpression of USP21 affected the protein levels of p53 and p21 in SW480 cells harboring mutant p53 (Fig. S3A), indicating that USP21 suppresses the protein expression of wild-type p53 rather than mutant p53. Luciferase reporter assays showed that USP21 knockdown enhanced p53 transcriptional activity, while overexpression of either wild-type USP21 or USP21 (C221A) mutant suppressed it (Fig. S3B, C). Consistently, USP21 knockdown upregulated, whereas USP21 overexpression downregulated, the mRNA levels of p53 target genes, including *Mdm2*, *p21*, *PUMA*, and *SESTRIN1* (Fig. S3D–G). Notably, neither knockdown nor overexpression of USP21 obviously affected p53 mRNA levels (Fig. S3D–S3G), indicating that USP21 inhibits p53 expression at the post-transcriptional level. In support of this, the inhibitory effect of USP21 on p53 protein stability was completely reversed by proteasome inhibition with MG132 (Fig. 4C, D). Cycloheximide chase assays revealed that USP21 knockdown extended the half-life of p53 (Fig. 4E and S3H), while USP21 overexpression showed the opposite effect (Fig. 4F and S3I). Furthermore, both immunoprecipitation and tandem-repeated ubiquitin-binding entities (TUBEs) pull-down assays demonstrated that knockdown of USP21 reduced p53 ubiquitination (Fig. 4G and S3J), whereas overexpression of USP21 enhanced it (Fig. 4H and S3K). Collectively, these data suggest that USP21 destabilizes p53 by promoting its ubiquitination and proteasomal degradation.

To determine whether USP21-mediated p53 suppression depends on Mdm2, we introduced two USP21 mutants into HCT116 cells: a truncation lacking Mdm2-binding ability (USP21 1–212) and a fragment retaining Mdm2-binding capability (USP21 213–565). Only the Mdm2-binding fragment (213–565) recapitulated wild-type USP21’s ability to suppress p53, while the binding-defective mutant (1–212) had no effect (Fig. 4I), indicating that the interaction with Mdm2 is crucial for USP21 to suppress p53 expression. We next examined whether USP21 requires Mdm2 to degrade p53. In HCT116 cells, overexpression of USP21 reduced p53 levels, but this effect was abolished upon Mdm2 knockdown (Fig. 4J). Consistent with this, USP21 overexpression enhanced p53 ubiquitination in control HCT116 cells, but not in Mdm2 knockdown HCT116 cells (Fig. 4K). To further validate Mdm2-dependence, we used Mdm2^−/−^p53^−/−^ mouse embryonic fibroblast (MEF) cells. USP21 alone did not alter p53 levels in the absence of Mdm2, but when co-expressed with Mdm2, USP21 promoted dose-dependent p53 degradation (Fig. S3L). Correspondingly, USP21 increased Mdm2-mediated p53 ubiquitination in a dose-dependent manner (Fig. S3M). To further investigate the requirement of USP7 in USP21-mediated p53 suppression, we employed rescue experiments. The dose-dependent reduction of p53 induced USP21 overexpression was abrogated upon simultaneous USP7 depletion (Fig. 4L). As expected, the increase in p53 ubiquitination induced by USP21 overexpression was abolished upon USP7 deletion (Fig. 4M). Together, these data strongly support that USP21 promotes p53 ubiquitination and degradation via USP7-Mdm2 axis.

To examine whether USP21 influences the Mdm2-p53 interaction, we performed co-immunoprecipitation assays. The results showed that USP21 did not associate with p53 (Fig. 4N). USP21 had no effect on the interaction between exogenously expressed Mdm2 and p53 (Fig. S3N). Furthermore, neither overexpression or knockdown of USP21 obviously altered the endogenous Mdm2-p53 binding (Fig. 4O, P). These findings collectively suggest that USP21 promotes p53 destabilization by increasing Mdm2 protein levels rather than modulating the Mdm2-p53 interaction.

### USP21 negatively regulates the tumor suppressive activity of p53

**Fig. 5:**
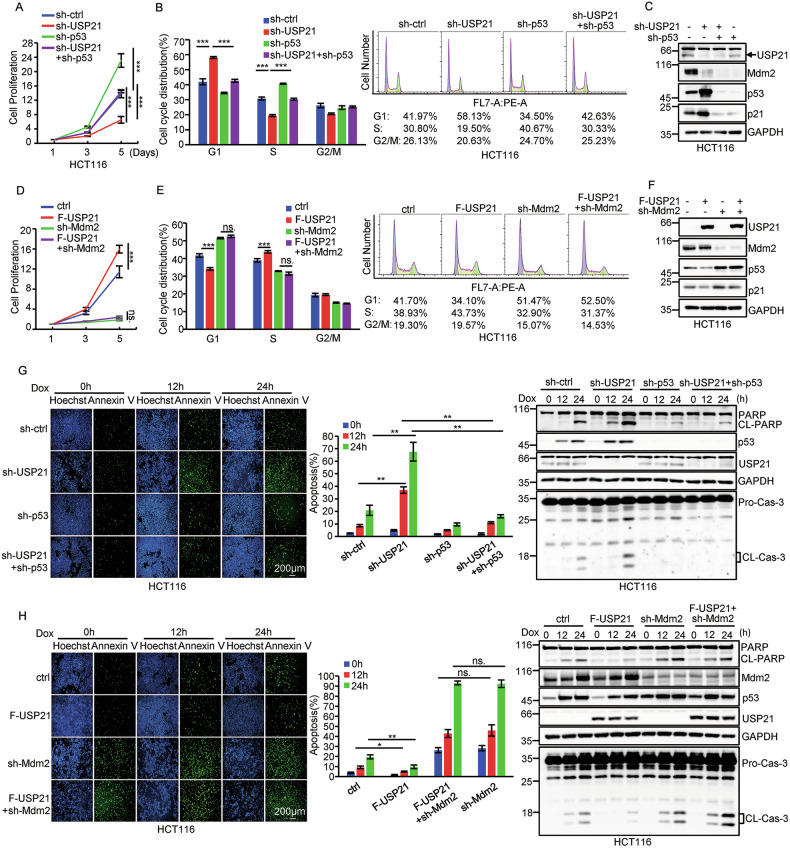
USP21 attenuates the tumor suppressive activity of p53. **A** Growth curves of HCT116 cells expressing control shRNA, USP21 shRNA, p53 shRNA, or USP21 shRNA plus p53 shRNA. Data shown are mean ± SD (*n* = 3). ***p < 0.001. **B** Cell cycle distribution of HCT116 cells expressing control shRNA, USP21 shRNA, p53 shRNA, or USP21 shRNA plus p53 shRNA. Data shown are mean ± SD (*n* = 3). ***p < 0.001. **C** Western blot analysis of lysates from HCT116 cells expressing control shRNA, USP21 shRNA, p53 shRNA, or USP21 shRNA plus p53 shRNA. **D** Growth curves of HCT116 cells expressing control, Flag-USP21, Mdm2 shRNA, or Flag-USP21 plus Mdm2 shRNA. Data shown are mean ± SD (*n* = 3). ***p < 0.001; ns, no significant difference. **E** Cell cycle distribution of HCT116 cells expressing control, Flag-USP21, Mdm2 shRNA, or Flag-USP21 plus Mdm2 shRNA. Data shown are mean ± SD (*n* = 3). ***p < 0.001; ns, no significant difference. **F** Western blot analysis of lysates from HCT116 cells expressing control, Flag-USP21, Mdm2 shRNA, or Flag-USP21 plus Mdm2 shRNA. **G** HCT116 cells expressing control shRNA, USP21 shRNA, p53 shRNA, or USP21 shRNA plus p53 shRNA were treated with 1 µg/mL doxorubicin (Dox) for the indicated periods of time. Cells were co-stained with Hoechst 33342 and Annexin V-FITC, and Annexin V-positive cells were quantified as apoptotic. Data are shown as mean ± SD (*n* = 3). **p < 0.01. Cell lysates were also subjected to Western blot analysis to detect cleaved PARP (CL-PARP) and cleaved caspase-3 (CL-Cas-3). **H** HCT116 cells expressing control, Flag-USP21, Mdm2 shRNA, or Flag-USP21 plus Mdm2 shRNA were treated with 1 µg/mL doxorubicin (Dox) for the indicated periods of time. Cells were co-stained with Hoechst 33342 and Annexin V-FITC, and Annexin V-positive cells were quantified as apoptotic. Data are shown as mean ± SD (*n* = 3). *p < 0.05; **p < 0.01; ns, no significant difference. Cell lysates were also subjected to Western blot analysis to detect cleaved PARP (CL-PARP) and cleaved caspase-3 (CL-Cas-3). Statistical analysis was performed using two-way ANOVA (**A**,**B**,**D**,**E**,**G**,**H**).

Given the destabilizing effect of USP21 on p53, we sought to determine whether USP21 regulates the tumor-suppressive activity of p53 by assessing its impact on cell proliferation, cell cycle progression, and apoptosis. In HCT116 and RKO cells, USP21 knockdown significantly suppressed cell proliferation and arrested G1/S transition (Fig. 5A–C and S4A–C). However, these effects were largely reversed by simultaneous knockdown of p53 (Fig. 5A–C and S4A–C). Conversely, ectopic expression of USP21 in both HCT116 and RKO cells promoted cell proliferation and accelerated G1/S progression (Fig. 5D–F and S4D–F). However, when p53 expression was induced by Mdm2 knockdown, USP21 overexpression failed to exert these effects (Fig. 5D–F and S4D–F). These findings suggest that USP21 facilitates cell proliferation and cell cycle progression by suppressing p53. To further evaluate the effect of USP21 on p53-mediated apoptosis, we treated HCT116 and RKO cells with the chemotherapeutic drug doxorubicin to activate p53. As expected, p53 knockdown abolished doxorubicin-induced apoptosis (Fig. 5G and S4G). Knockdown of USP21 sensitized cells to doxorubicin-induced apoptosis; however, simultaneous knockdown of p53 reversed this effect (Fig. 5G and S4G). Moreover, ectopic expression of USP21 reduced the sensitivity of doxorubicin-induced apoptosis in control cells, but not in Mdm2 knockdown cells, where p53 was upregulated (Fig. 5H and S4H). Collectively, these data support that USP21 negatively regulates the tumor-suppressive activity of p53.

### USP21 promotes colorectal cancer progression via inhibition of p53

**Fig. 6:**
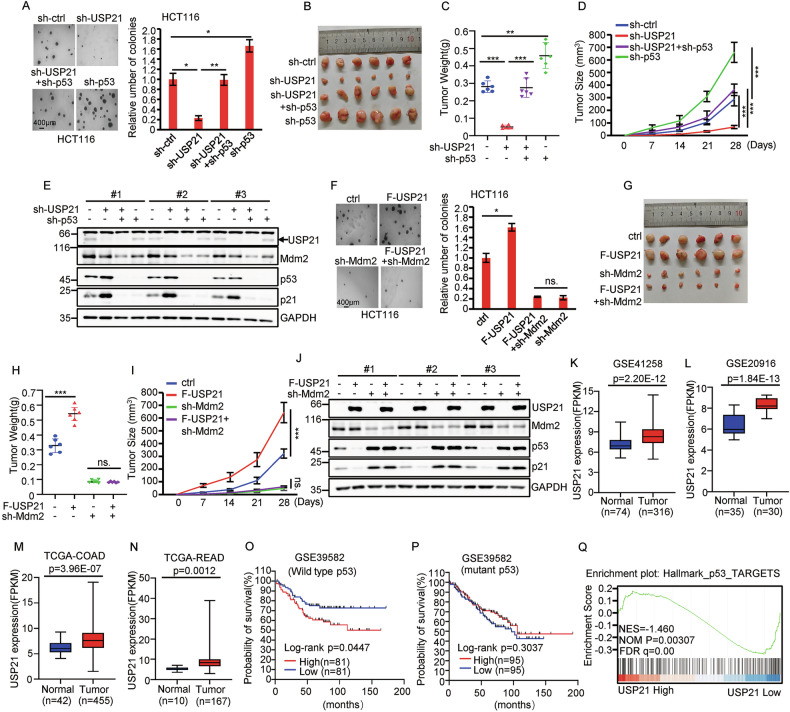
USP21 promotes colorectal cancer progression via inhibition of p53. **A** HCT116 cells expressing control shRNA, USP21 shRNA, p53 shRNA, or USP21 shRNA plus p53 shRNA were assayed for their ability to form colonies in soft agar. Data are shown as mean ± SD (*n* = 3). *p < 0.05, **p < 0.01. **B–E** A total of 2 × 10^6^ HCT116 cells expressing control shRNA, USP21 shRNA, p53 shRNA, or USP21 shRNA plus p53 shRNA were injected into the flanks of nude mice (*n* = 6 per group). Tumor xenografts were harvested 28 days after injection (**B**). Tumor weights were measured (**C**), and tumor size was monitored at the indicated time points (**D**). **p < 0.01; ***p < 0.001. Tumor lysates were analyzed by western blotting (**E**). **F** HCT116 cells expressing control, Flag-USP21, Mdm2 shRNA, or Flag-USP21 plus Mdm2 shRNA were assayed for their ability to form colonies in soft agar. Data are shown as mean ± SD (*n* = 3). *p < 0.05; ns, no significant difference. **G–J** A total of 2 × 10^6^ HCT116 cells expressing control, Flag-USP21, Mdm2 shRNA, or Flag-USP21 plus Mdm2 shRNA were injected into the flanks of nude mice (*n* = 6 per group). Tumor xenografts were harvested 28 days after injection (**G**). Tumor weights were measured (**H**), and tumor size was monitored at the indicated time points (**I**). ***p < 0.001. Tumor lysates were analyzed by western blotting (**J**). **K** Box plot showing the upregulation of USP21 in tumor tissues compared to adjacent normal tissues in the GEO colorectal cancer dataset (GSE41258). **L** Box plot showing the upregulation of USP21 in tumor tissues compared to adjacent normal tissues in the GEO colorectal cancer dataset (GSE20916). **M** Box plot showing the upregulation of USP21 in TCGA colon adenocarcinoma (COAD) compared to adjacent normal tissues. **N** Box plot showing the upregulation of USP21 in TCGA rectum adenocarcinoma (READ) compared to adjacent normal tissues. **O**,**P** Kaplan-Meier analysis of the impact of USP21 expression on overall survival of colorectal cancer patients with wild-type p53 (**O**) and mutant p53 (**P**), based on GEO dataset (GSE39582). **Q** Gene Set Enrichment Analysis (GSEA) of wild-type p53 colorectal cancer samples from GSE39582 showing an upregulation of p53 target genes in the USP21 low-expression group. Statistical analysis was performed using one-way ANOVA (**A**,**C**,**F**,**H**), two-way ANOVA (**D**,**I**), two-tailed Student’s t-test (**K**,**L**,**M**,**N**), or log-rank test (**O**,**P**).

We next investigated whether USP21 exerts its oncogenic function by suppressing p53. Soft agar colony formation assays showed that knockdown of USP21 significantly impaired anchorage-independent growth of HCT116 cells, an effect rescued by concurrent p53 knockdown (Fig. 6A). Similarly, in a xenograft mouse model, USP21 knockdown markedly suppressed tumor growth in vivo, and this suppression was reversed upon p53 depletion (Fig. 6B–E). To determine whether Mdm2 mediates USP21’s oncogenic effects, we examined the consequences of Mdm2 knockdown in USP21-overexpressing cells. While overexpression of USP21 in HCT116 cells enhanced colony formation in soft agar, this effect was abolished when Mdm2 was silenced (Fig. 6F). In vivo, the promoting effect of USP21 overexpression on xenograft tumor growth was completely lost upon Mdm2 knockdown, which coincided with p53 induction (Fig. 6G–J). Together, these findings suggest that USP21 promotes tumor progression by suppressing p53 in an Mdm2-dependent manner.

To further evaluate the clinical significance of USP21 in colorectal cancer, we analyzed its expression across multiple colorectal cancer transcriptomic datasets. USP21 was significantly upregulated in colorectal cancer tissues compared to normal tissues in the GSE41258, GSE20916, TCGA-COAD, and TCGA-READ cohorts (Fig. 6K–N). Consistent with the specific regulation of wild-type p53 by USP21 (Fig. 4A, B and S3A), Kaplan–Meier survival analysis using the GSE39582 dataset showed that high USP21 expression was associated with poor overall survival in patients with wild-type p53 colorectal cancer, but not in those with p53-mutated colorectal cancer (Fig. 6O, P). Furthermore, Gene Set Enrichment Analysis (GSEA) of wild-type p53 colorectal cancer samples from GSE39582 revealed a significant upregulation of p53 target genes in the USP21 low-expression group (Fig. 6Q). Taken together, these findings suggest that USP21 promotes colorectal cancer progression via inhibition of p53.

## Discussion

p53 is one of the most important tumor suppressors, and its inactivation represents a key event in tumorigenesis [37]. The current study identifies the deubiquitinating enzyme USP21 as a negative regulator of p53. Mechanistically, USP21 strengthens the USP7-Mdm2 interaction, leading to Mdm2 stabilization and subsequent p53 degradation. Functionally, USP21 facilitates colorectal cancer progression by suppressing p53 activity. Thus, USP21 plays a crucial role in regulating the Mdm2-p53 pathway.

Mdm2 is well-established as a negative regulator of p53, but it also undergoes autoubiquitination and rapid degradation [10, 11]. Beyond this self-regulatory mechanism, Mdm2 is further destabilized by several E3 ubiquitin ligases, including PCAF, SCF^β-TrCP^, FBXO31, XIAP, and NAT10 [38–42]. Conversely, Mdm2 is stabilized by multiple deubiquitinating enzymes such as USP7, USP2a, and USP15 [19, 43, 44]. We here show that USP21 interacts with and stabilizes Mdm2. This stabilization does not involve the inhibition of Mdm2 autoubiquitination, as USP21 still upregulates the E3 ligase-deficient Mdm2 mutant (C464A). USP21 typically functions as a deubiquitinating enzyme in diverse pathways, including Wnt activation via TCF7 deubiquitination, cell cycle regulation in breast cancer via FOXM1 stabilization, and colorectal cancer metastasis via EGFR and Fra-1 modulation [24, 27, 31, 45]. However, our study uncovers a previously unappreciated deubiquitinase activity-independent role for USP21: it acts as a scaffold to enhance the USP7-Mdm2 interaction, facilitating USP7-mediated deubiquitination and stabilization of Mdm2.

USP7 comprises three functional domains: an N-terminal TRAF-like domain, a central catalytic domain, and five C-terminal ubiquitin-like (UBL) domains. As a critical regulator of the Mdm2-p53 axis, USP7’s activity and substrate specificity are tightly controlled by diverse binding partners. For instance, TSPYL5, EBNA1, and MLF2 inhibit USP7 by blocking its interaction with p53, thereby accelerating p53 ubiquitination [46–48]. Conversely, ABRO1 promotes the USP7-p53 interaction to enhance p53 deubiquitination [49]. DAXX functions as an adapter, facilitating USP7-Mdm2 association to stabilize Mdm2 and promote p53 degradation [50]. Moreover, RALY binds to USP7 C-terminal UBL domains and stimulates the deubiquitinating activity toward Mdm2 [51]. In this study, we show that USP21, USP7, and Mdm2 form a ternary complex in which USP21 potentiates the USP7-Mdm2 interaction. This enhancement promotes Mdm2-mediated p53 degradation. These findings reveal that USP7’s substrate preference toward either Mdm2 or p53 is precisely modulated by its distinct binding partners, indicating the complexity of the regulation of the Mdm2-p53 pathway.

Accumulating evidence implicates USP21 as an oncogenic factor in a variety of human cancers, including breast cancer, hepatocellular carcinoma, and lung cancer [52–54]. Consistent with these findings, USP21 is upregulated in colorectal cancer tissues compared to adjacent normal tissues. Functionally, USP21 negatively regulates the tumor suppressive activity of p53 and promotes colorectal cancer progression via p53 inhibition. Analysis of colorectal cancer patient samples with wild-type p53 reveals a significant inverse correlation between USP21 expression and p53 target gene levels, highlighting the physiological relevance of USP21-mediated p53 suppression in colorectal cancer. Moreover, high expression of USP21 is associated with poor prognosis in patients with wild-type colorectal cancer but not those with p53-mutated colorectal cancer. Taken together, our study establishes USP21 as an important regulator of the Mdm2-p53 pathway and proposes it as a potential therapeutic target for colorectal cancer harboring wild-type p53.

## Materials and methods

### Reagents and antibodies

All commercial reagents and antibodies were obtained as follows: anti-Flag M2 affinity agarose gel, cycloheximide (20 μg/mL), polybrene (10 μg/mL), Hoechst 33342 (1 μg/mL), N-ethylmaleimide (2 mM), doxorubicin (1 μg/mL), and Annexin V-FITC from Sigma-Aldrich (Burlington, MA, USA); MG132 (20 μM) and complete EDTA-free protease inhibitor cocktail from APExBIO (Houston, TX, USA); Protein A/G agarose beads from Thermo Fisher Scientific (Waltham, MA, USA); TRIzol reagent and Lipofectamine 2000 from Invitrogen (Waltham, MA, USA); Glutathione Sepharose beads from Beyotime Biotechnology (Shanghai, China); and Seaplaque low melting temperature agarose from Lonza (Basel, Switzerland). Primary antibodies included: anti-USP21 (17856-1-AP, 1:1500, Proteintech, Wuhan, China), anti-USP7 (A300-033A, 1:2000, Bethyl, Montgomery, TX, USA), anti-GAPDH (GB12002-100, 1:5000, Servicebio, Wuhan, China), anti-GAPDH (sc-166545, 1:10000, Santa Cruz, Dallas, TX, USA), anti-GFP (Santa Cruz, sc-9996, 1:1000), anti-p53 (Santa Cruz, sc-126, 1:1000), anti-p21 (Sigma-Aldrich, P1484, 1:1000), anti-Mdm2 (Santa Cruz, sc-965, 1:500), anti-PARP (Santa Cruz, sc-8007, 1:1500), anti-ubiquitin (#3936, 1:500, Cell Signaling, Boston, MA, USA), anti-caspase-3 (Cell Signaling, #9662, 1:1000), anti-HA (Sigma-Aldrich, H9658, 1:3000), and anti-Flag (Sigma-Aldrich, F3165, 1:3000). HRP-conjugated secondary antibodies were anti-mouse IgG (115-035-062, 1:10000) and anti-rabbit IgG (111-035-144, 1:10000) from Jackson ImmunoResearch (West Grove, PA, USA).

### Cell culture

HCT116 (CCL-247), RKO (CRL-2577), HEK293T (CRL-3216), and H1299 (CRL-5803) cells were obtained from the American Type Culture Collection (ATCC, Manassas, Virginia, USA). The Mdm2^−/−^p53^−/−^ MEF cell line was generated from wild-type mouse embryonic fibroblasts (MEFs) using CRISPR/Cas9-mediated knockout of both *Mdm2* and *Tp53* genes. HCT116, RKO, HEK293T, and Mdm2^−/−^p53^−/−^ MEF cells were cultured in Dulbecco’s Modified Eagle Medium (Gibco, Grand Island, NY, USA) supplemented with 10% fetal bovine serum and 1% penicillin/streptomycin. H1299 cells were maintained in RPMI-1640 medium (Gibco) supplemented with 10% fetal bovine serum and 1% penicillin/streptomycin. All cell lines have been authenticated in the past 3 years by short tandem repeat (STR) genotyping and were routinely tested for mycoplasma contamination.

### Identification of USP21 as an Mdm2-interacting protein

HCT116 cells were cross-linked with 0.2% formaldehyde. The reaction was terminated by adding 0.15 M glycine (pH 7.4). Subsequently, cells were lysed in RIPA buffer (50 mM Tris-HCl, pH 7.5, 150 mM NaCl, 1 mM EDTA, 0.5% Triton X-100, 0.5% NP-40, 1% sodium deoxycholates, 0.1% SDS, and 20 μM MG132) supplemented with 1× protease inhibitor cocktail. Following sonication, the lysates were pre-cleared with protein A/G-coupled agarose beads. Immunoprecipitation was then performed with an anti-Mdm2 antibody for 8 h at 4 °C. After three washes, bead-bound proteins were eluted with elution buffer (10 mM Tris-HCl, pH 7.5, 100 mM NaCl, 2.5 mM MgCl_2_, and 0.4% SDS) at room temperature for 30 min. The eluates were subsequently analyzed by mass spectrometry using a Thermo Q Exactive Orbitrap mass spectrometer at the National Facility for Protein Science (Shanghai Advanced Research Institute, Chinese Academy of Science), and the data were analyzed with Proteome Discoverer software (version 2.4).

### Lentivirus production and infection

Lentiviruses expressing Flag-USP21 or the indicated proteins were generated by transfecting HEK293T cells with pLVX-based constructs, psPAX2, and pMD2.G using Polyethylenimine “Max” (Polysciences, Warrington, PA, USA). For shRNA-expressing lentiviruses, HEK293T cells were transfected with PLKO.1-shRNA constructs along with pREV, pGag/Pol/PRE, and pVSVG. Control viruses were produced using either empty pLVX vector or PLKO.1 containing a scrambled shRNA. Six hours after transfection, the medium was replaced, and cells were cultured for an additional 36 h. Viral supernatants were collected, filtered through a 0.45-μm PVDF filter (Millipore, Darmstadt, Germany), and used to infect target cells in the presence of 10 μg/mL polybrene. Functional assays were conducted 48 h post-infection. The shRNA target sequences are provided in Supplementary Table 2.

### Real-time RT-PCR

Total RNA was isolated using Trizol reagent (Invitrogen). cDNA synthesis was performed with 1 μg RNA using HiScript II Q RT SuperMix (R222-01, Vazyme, Nanjing, China) according to the manufacturer’s protocol. Quantitative real-time PCR was carried out with SYBR Premix Ex Taq (TaKaRa, Shiga, Japan) on the StepOnePlus real-time PCR System (Thermo Fisher Scientific). GAPDH was used as an internal control. Relative gene expression was calculated using the 2^-ΔΔCt^ method. The primer sequences are listed in Supplementary Table 2.

### Co-immunoprecipitation

HCT116 or other indicated cell lines were treated with 20 μM MG132 for 6 h before they were lysed in IP buffer (50 mM Tris-HCl, pH 7.4, 150 mM NaCl, 1.5 mM MgCl_2_, 1 mM EDTA, 0.5% NP-40, 0.5% Triton X-100, 10% glycerol) supplemented with protease inhibitor cocktail. After gentle sonication, cell lysates were pre-cleared with protein A/G agarose beads for 4 h, and then immunoprecipitated with specified antibodies. Both immunoprecipitates and whole cells lysates were resolved by SDS-PAGE and analyzed by western blotting.

### Dual-luciferase reporter assay

To evaluate the effect of USP21 on p53 transcriptional activity, HCT116 cells transduced with the indicated lentiviruses were co-transfected with pGL3 control, pGL3-p21, or pGL3-NOXA reporter plasmids, along with a Renilla luciferase plasmid for normalization. Twenty-four hours after transfection, firefly and Renilla luciferase activities were measured using the Dual-Luciferase Reporter Assay System (Promega, Madison, WI, USA).

### In vivo ubiquitination assay

Cells were lysed by boiling for 10 min in denaturing buffer (150 mM Tris-HCl, pH 8.0, 1% SDS, 30% glycerol). Lysates were diluted 5-fold with buffer A (50 mM Tris-HCl, pH 8.0, 150 mM NaCl, 1% NP-40, 2 mM N-ethylmaleimide, protease inhibitor cocktail) and subjected to immunoprecipitation with anti-p53 or anti-Mdm2 antibodies at 4 °C overnight, followed by western blot analysis. Alternatively, cells were lysed in denaturation buffer (6 M guanidine-HCl, pH 8.0, 0.1 M Na_2_HPO4/NaH_2_PO_4_, 10 mM imidazole) and incubated with Ni-NTA agarose beads at room temperature for 4 h prior to western blotting.

### Protein expression and purification

The DNA sequences encoding USP21, Mdm2, or TUBEs (consisting of four tandem repeats of the UBA domains of human HR23A) were cloned into the pGEX-6P-1 vector and transformed into *Escherichia coli* BL21 (DE3) cells. Recombinant GST-tagged proteins were induced with 0.2 mM IPTG at 16 °C for 20 h and purified by glutathione affinity chromatography.

To purify Flag-tagged proteins (USP21, Mdm2, and USP7), the corresponding pRK5-based overexpressing plasmids were transfected into HEK293T cells. Proteins were immunoprecipitated using anti-Flag M2 beads, washed with lysis buffer containing increasing KCl concentrations (0.25, 0.5, and 1 M) to remove non-specific binding, and eluted with 3×Flag peptide.

### GST and TUBE pull-down assays

For GST pull-down assay, GST-tagged proteins immobilized on glutathione beads were incubated with target proteins in buffer (50 mM Tris-HCl, pH 7.4, 150 mM NaCl, 0.5% Triton X-100) at 4 °C for 4 h. For TUBE pull-down assay, HCT116 cells pre-treated with 20 μM MG132 for 6 h were lysed by gentle sonication in lysis buffer (50 mM Tris-HCl, pH 7.4, 150 mM NaCl, 1.5 mM MgCl_2_, 1 mM EDTA, 0.5% NP-40, 0.5% Triton X-100, 10% glycerol, 2 mM N-ethylmaleimide, 20 μM MG132). Lysates were incubated with glutathione beads immobilized with GST-TUBE proteins at 4 °C for 4 h. For both assays, input samples and bead-bound proteins were resolved by SDS-PAGE and analyzed by western blotting.

### Colony formation in soft agar

HCT116 cells were infected with the indicated lentiviruses. Forty-eight hours post-infection, 1 × 10^4^ cells were suspended in DMEM containing 10% FBS and 0.3% Seaplaque low-melting temperature agarose and overlaid onto a base layer of solidified DMEM/10% FBS/0.6% agarose. Following a 12-day incubation at 37 °C, cells were fixed and stained with crystal violet. Colonies were then counted under an Olympus IX73 microscope (Tokyo, Japan).

### Cell cycle analysis

HCT116 and RKO cells were infected with the indicated lentiviruses. Forty-eight hours post-infection, cells were fixed in 70% ethanol at −20 °C overnight and subsequently stained with propidium iodide. Cell cycle distribution was then assessed by flow cytometry.

### Apoptosis assay

HCT116 and RKO cells were infected with the indicated lentiviruses and treated with doxorubicin 48 h post-infection. Apoptosis was assessed by co-staining with Annexin V-FITC and Hoechst 33342, with Annexin V-positive cells quantified as apoptotic. Cell lysates were also subjected to western blot analysis using anti-PARP and anti-Caspase 3 antibodies.

### Xenograft mouse model

For the xenograft mouse model, 2 × 10^6^ lentivirus-transduced HCT116 cells were subcutaneously injected into the flanks of 4-week-old male BALB/c nude mice (*n* = 6 for each group). Tumor volume was measured every week using a caliper and calculated with the equation: volume = length × width² × 0.52. After 4 weeks, mice were sacrificed, and tumors were excised and weighed. Tumor lysates were also analyzed by western blotting. The experimentalists were blinded to the information about tumor tissues when tumors were excised and weighed.

### Reproducibility

All experiments were repeated at least three times with similar results. The presented blots and images are representative of three independent experiments.

### Statistical analysis

The sample size for each experiment was determined based on common practices in the field and animal welfare considerations, and is stated in the relevant figure legends. For animal studies, mice were randomly assigned to experimental groups. All data collection and analysis were performed with the investigators blinded to group allocation. Data analysis was conducted using Microsoft Excel (Redmond, WA, USA) and GraphPad Prism software (GraphPad Software Inc., San Diego, CA, USA). Data variance within each group or between groups was assessed. Statistical significance was evaluated using either two-tailed Student’s t-test, one-way ANOVA, or two-way ANOVA, as appropriate. The log-rank test was used to calculate the Kaplan–Meier survival curve. Data are presented with corresponding p-values, with statistical significance set at p < 0.05. Significance levels are denoted as follows: *p < 0.05, **p < 0.01, and ***p < 0.001; ns. indicates no significance.

## Supplementary information

Supplementary Table 1

Supplementary Table 2

Supplementary Figures

Original data for western blot

## Data Availability

The data that support the findings of this study are available from the corresponding author upon reasonable request. The original data of western blot are provided in the Supplementary information.
