# Supplementary material for: USP21 functions as an oncogenic regulator of the Mdm2-p53 axis in colorectal cancer
Source: Cell Death Discov. 2026 May 22;12:305. doi: 10.1038/s41420-026-03170-3 (PMC13370000; doi:10.1038/s41420-026-03170-3)
Supplement: Supplementary file 3 — Supplementary Figures [file 41420_2026_3170_MOESM3_ESM.pdf]

Figure legends

Figure S1. Related to Figure 2.

(A) HEK293T cells were transfected with either HA-Mdm2 alone or together with increasing amounts of Flag-USP21 mutants. Twenty-four hours later, cell lysates were analyzed by western blotting.

(B) The blots from Figure 2H were quantified using Image J software and the ratio of Mdm2 to GAPDH was calculated. Data are shown as mean ± SD (n=3). ***p < 0.001.

(C) The blots from Figure 2I were quantified using Image J software and the ratio of Mdm2 to GAPDH was calculated. Data are shown as mean ± SD (n=3). ***p < 0.001.

(D) The blots from Figure 2J were quantified using Image J software and the ratio of Mdm2 to GAPDH was calculated. Data are shown as mean ± SD (n=3). ***p < 0.001. Statistical analysis was performed using two-way ANOVA (B, C, D).

Figure S2. Related to Figure 3.

(A) Lysates from HEK293T cells expressing HA-USP21 alone or HA-USP21 plus the indicated Flag-tagged USP7 mutants were subjected to immunoprecipitation analysis.

(B) Lysates from HEK293T cells expressing Flag-USP7 alone or Flag-USP7 plus the indicated HA-tagged USP21 mutants were subjected to immunoprecipitation analysis.

(C) Lysates from HEK293T cells expressing Flag-USP7, Flag-USP7 plus GFP-USP21 (213-390), or Flag-USP7 plus GFP-USP21 (391-565) were subjected to immunoprecipitation analysis.

(D) Lysates from HEK293T cells expressing Flag-Mdm2, Flag-Mdm2 plus GFP-USP21 (213-390), or Flag-Mdm2 plus GFP-USP21 (391-565) were subjected to immunoprecipitation analysis.

Figure S3. Related to Figure 4.

(A) Western blot analysis of lysates from SW480 cells with USP21 knockdown or overexpression.

(B) HCT116 cells expressing control shRNA, USP21 shRNA#1, or USP21 shRNA#2 were transfected with the indicated luciferase reporter constructs plus a Renilla

luciferase plasmid. Luciferase activity was measured 24 hours post-transfection. Data are shown as mean ± SD (n=3). **p < 0.01; ns, no significant difference.

(C) HCT116 cells expressing control, Flag-USP21, or Flag-USP21(C221A) were transfected with the indicated luciferase reporter constructs plus a Renilla luciferase plasmid. Luciferase activity was measured 24 hours post-transfection. Data are shown as mean ± SD (n=3). **p < 0.01; ns, no significant difference.

(D) Real-time RT-PCR analysis of total RNA from HCT116 cells expressing control shRNA or USP21 shRNA. Data are shown as mean ± SD (n=3). **p < 0.01; ns, no significant difference.

(E) Real-time RT-PCR analysis of total RNA from HCT116 cells expressing control or Flag-USP21. Data are shown as mean ± SD (n=3). *p < 0.05; **p < 0.01; ns, no significant difference.

(F) Real-time RT-PCR analysis of total RNA from RKO cells expressing control shRNA or USP21 shRNA. Data are shown as mean ± SD (n=3). **p < 0.01; ***p < 0.001; ns, no significant difference.

(G) Real-time RT-PCR analysis of total RNA from RKO cells expressing control or Flag-USP21. Data are shown as mean ± SD (n=3). *p < 0.05; **p < 0.01; ***p < 0.001; ns, no significant difference.

(H) The blots from Figure 4E were quantified using Image J software and the ratio of p53 to GAPDH was calculated. Data are shown as mean ± SD (n=3). ***p < 0.001.

(I) The blots from Figure 4F were quantified using Image J software and the ratio of p53 to GAPDH was calculated. Data are shown as mean ± SD (n=3). ***p < 0.001.

(J) HCT116 cells expressing control shRNA or USP21 shRNA were treated with 20 μM MG132 for 6 hours. Cell lysates were then incubated with GST-TUBEs (HR23A) immobilized on glutathione beads. Both input and bead-bound proteins were analyzed by western blotting.

(K) HCT116 cells expressing control or Flag-USP21 were treated with 20 μM MG132 for 6 hours. Cell lysates were then incubated with GST-TUBEs (HR23A) immobilized on glutathione beads. Both input and bead-bound proteins were analyzed by western blotting.

(L) Mdm2–/–p53–/– MEF (DKO) cells were transfected with Flag-p53, Flag-Mdm2, and increasing amounts of HA-USP21 as indicated. Twenty-four hours later, cell lysates were analyzed by western blotting.
(M) Mdm2–/–p53–/– MEF (DKO) cells were transfected with Flag-p53, HA-Mdm2, His–ubiquitin, and increasing amounts of HA-USP21 as indicated. Twenty-four hours later, cells were treated with 20 μM MG132 for 6 hours, followed by an in vivo ubiquitination assay.
(N) HEK293T cells were transfected with GFP-p53, Flag-Mdm2, and increasing amounts of HA-USP21 as indicated. Twenty-four hours later, cells were treated with 20 μM MG132 for an additional 6 hours, followed by immunoprecipitation analysis.
Statistical analysis was performed using one-way ANOVA (B, C), two-tailed Student's t-test (D, E, F, G), or two-way ANOVA (H, I).

Figure S4. Related to Figure 5.
(A) Growth curves of RKO cells expressing control shRNA, USP21 shRNA, p53 shRNA, or USP21 shRNA plus p53 shRNA. Data shown are mean ± SD (n = 3). ***$p < 0.001$.
(B) Cell cycle distribution of RKO cells expressing control shRNA, USP21 shRNA, p53 shRNA, or USP21 shRNA plus p53 shRNA. Data shown are mean ± SD (n = 3). ***$p < 0.001$.
(C) Western blot analysis of lysates from RKO cells expressing control shRNA, USP21 shRNA, p53 shRNA, or USP21 shRNA plus p53 shRNA.
(D) Growth curves of RKO cells expressing control, Flag-USP21, Mdm2 shRNA, or Flag-USP21 plus Mdm2 shRNA. Data shown are mean ± SD (n = 3). ***$p < 0.001$; ns, no significant difference.
(E) Cell cycle distribution of RKO cells expressing control, Flag-USP21, Mdm2 shRNA, or Flag-USP21 plus Mdm2 shRNA. Data shown are mean ± SD (n = 3). ***$p < 0.001$; ns, no significant difference.
(F) Western blot analysis of lysates from RKO cells expressing control, Flag-USP21, Mdm2 shRNA, or Flag-USP21 plus Mdm2 shRNA.

(G) RKO cells expressing control shRNA, USP21 shRNA, p53 shRNA, or USP21 shRNA plus p53 shRNA were treated with 1 μg/mL doxorubicin (Dox) for the indicated periods of time. Cells were co-stained with Hoechst 33342 and Annexin V-FITC, and Annexin V-positive cells were quantified as apoptotic. Data are shown as mean ± SD ($n = 3$). **$p < 0.01$, ***$p < 0.001$. Cell lysates were also subjected to Western blot analysis to detect cleaved PARP (CL-PARP) and cleaved caspase-3 (CL-Cas-3).

(H) RKO cells expressing control, Flag-USP21, Mdm2 shRNA, or Flag-USP21 plus Mdm2 shRNA were treated with 1 μg/mL doxorubicin (Dox) for the indicated periods of time. Cells were co-stained with Hoechst 33342 and Annexin V-FITC, and Annexin V-positive cells were quantified as apoptotic. Data are shown as mean ± SD ($n = 3$). *$p < 0.05$; ns, no significant difference. Cell lysates were also subjected to Western blot analysis to detect cleaved PARP (CL-PARP) and cleaved caspase-3 (CL-Cas-3).

Statistical analysis was performed using two-way ANOVA (A, B, D, E, G, H).

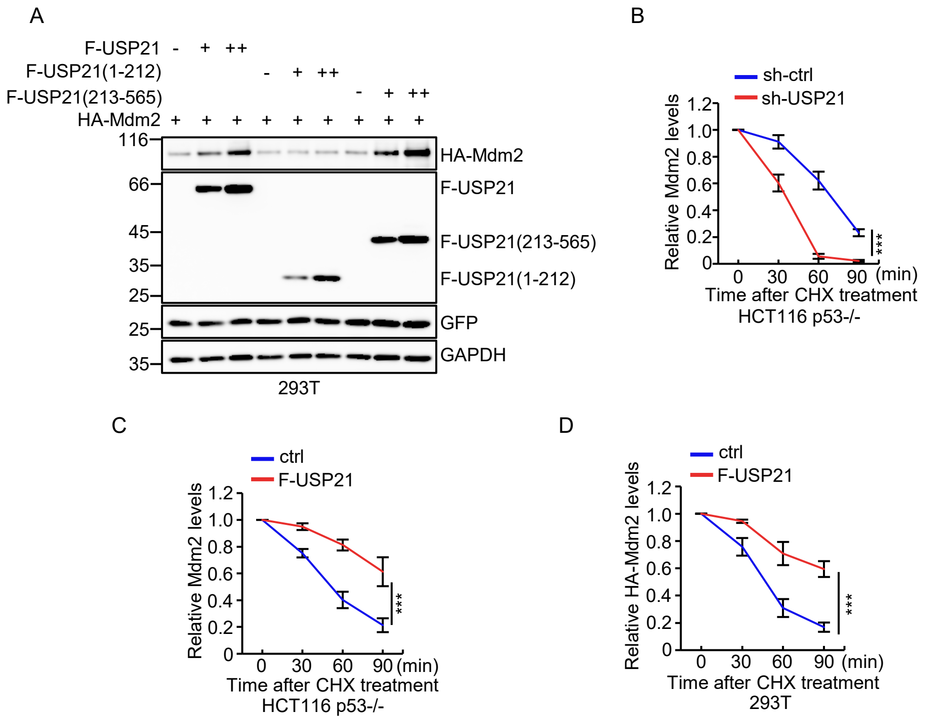
A
F-USP21 - + ++
F-USP21(1-212) - + ++
F-USP21(213-565) - + ++
HA-Mdm2 + + + + + + + + +
116
66
45
35
25
25
35
HA-Mdm2
F-USP21
F-USP21(213-565)
F-USP21(1-212)
GFP
GAPDH
293T
B
sh-ctrl
sh-USP21
Relative Mdm2 levels
1.2
1.0
0.8
0.6
0.4
0.2
0
0 30 60 90 (min)
***
Time after CHX treatment
HCT116 p53-/-
C
ctrl
F-USP21
Relative Mdm2 levels
1.2
1.0
0.8
0.6
0.4
0.2
0
0 30 60 90 (min)
***
Time after CHX treatment
HCT116 p53-/-
D
ctrl
F-USP21
Relative HA-Mdm2 levels
1.2
1.0
0.8
0.6
0.4
0.2
0
0 30 60 90 (min)
***
Time after CHX treatment
293T

Figure S2

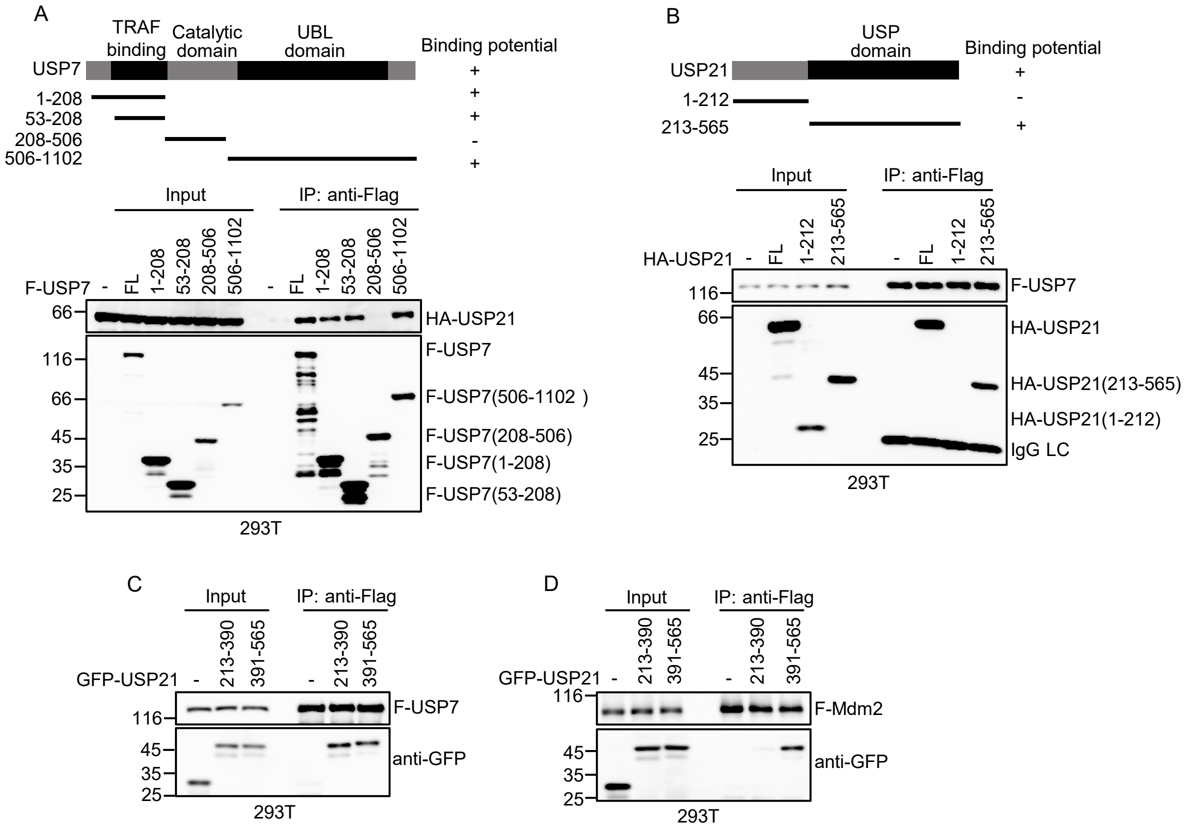
A
TRAF binding
Catalytic domain
UBL domain
Binding potential
USP7 +
1-208 +
53-208 +
208-506 -
506-1102 +
Input
IP: anti-Flag
F-USP7
-
FL
1-208
53-208
208-506
506-1102
66
116
45
35
25
HA-USP21
F-USP7
F-USP7(506-1102)
F-USP7(208-506)
F-USP7(1-208)
F-USP7(53-208)
293T
B
USP domain
Binding potential
USP21 +
1-212 -
213-565 +
Input
IP: anti-Flag
HA-USP21
-
FL
1-212
213-565
116
66
45
35
25
F-USP7
HA-USP21
HA-USP21(213-565)
HA-USP21(1-212)
IgG LC
293T
C
Input
IP: anti-Flag
GFP-USP21
-
213-390
391-565
116
45
35
25
F-USP7
anti-GFP
293T
D
Input
IP: anti-Flag
GFP-USP21
-
213-390
391-565
116
45
35
25
F-Mdm2
anti-GFP
293T

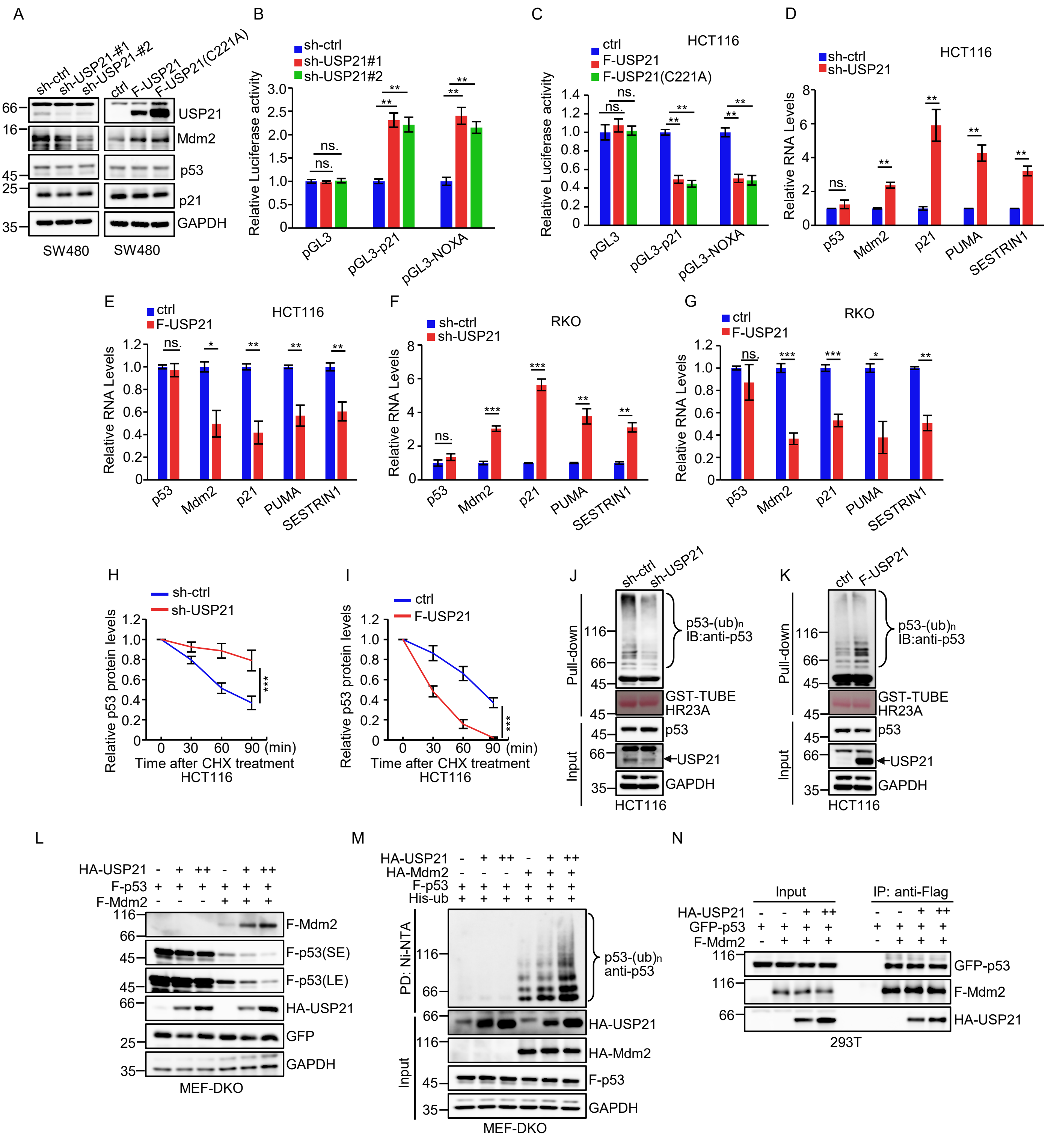
A
sh-ctrl
sh-USP21-#1
sh-USP21-#2
ctrl
F-USP21
F-USP21(C221A)
USP21
Mdm2
p53
p21
GAPDH
SW480
SW480
B
sh-ctrl
sh-USP21#1
sh-USP21#2
Relative Luciferase activity
pGL3
pGL3-p21
pGL3-NOXA
C
HCT116
ctrl
F-USP21
F-USP21(C221A)
D
HCT116
sh-ctrl
sh-USP21
Relative RNA Levels
p53
Mdm2
p21
PUMA
SESTRIN1
E
HCT116
F
RKO
G
RKO
H
Relative p53 protein levels
Time after CHX treatment
HCT116
I
J
p53-(ub)n
IB:anti-p53
Pull-down
GST-TUBE
HR23A
Input
K
L
HA-USP21
F-p53
F-Mdm2
F-p53(SE)
F-p53(LE)
GFP
MEF-DKO
M
His-ub
PD: Ni-NTA
HA-Mdm2
N
Input
IP: anti-Flag
GFP-p53
293T

Figure S4

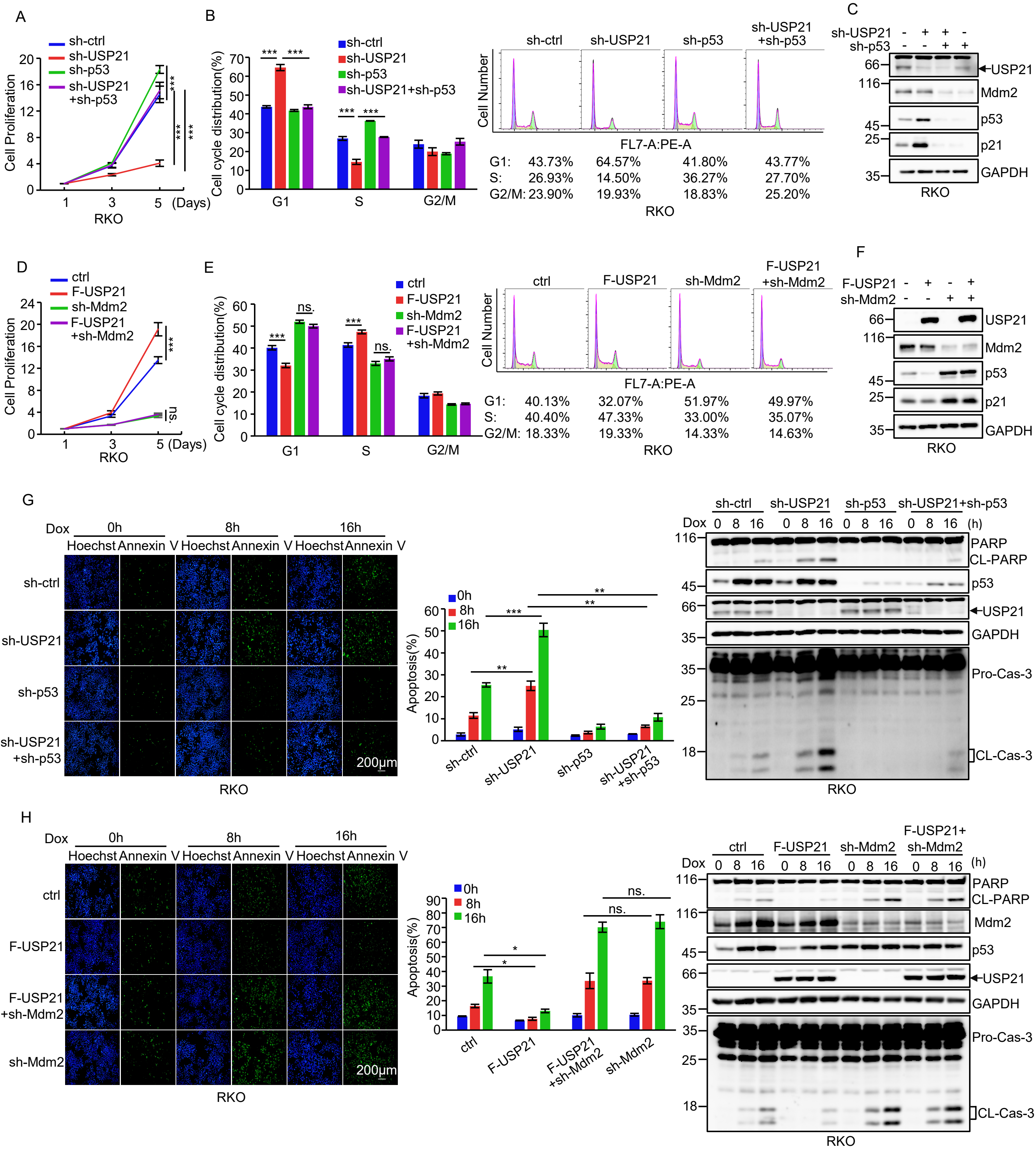

A
sh-ctrl
sh-USP21
sh-p53
sh-USP21 +sh-p53
Cell Proliferation
(Days)
RKO
B
Cell cycle distribution(%)
G1
S
G2/M
sh-ctrl
sh-USP21
sh-p53
sh-USP21
+sh-p53
Cell Number
FL7-A:PE-A
G1: 43.73% 64.57% 41.80% 43.77%
S: 26.93% 14.50% 36.27% 27.70%
G2/M: 23.90% 19.93% 18.83% 25.20%
RKO
C
USP21
Mdm2
p53
p21
GAPDH
RKO
D
ctrl
F-USP21
sh-Mdm2
F-USP21 +sh-Mdm2
Cell Proliferation
(Days)
RKO
E
Cell cycle distribution(%)
ctrl
F-USP21
sh-Mdm2
F-USP21
+sh-Mdm2
Cell Number
FL7-A:PE-A
G1: 40.13% 32.07% 51.97% 49.97%
S: 40.40% 47.33% 33.00% 35.07%
G2/M: 18.33% 19.33% 14.33% 14.63%
RKO
F
USP21
Mdm2
p53
p21
GAPDH
RKO
G
Dox
0h
8h
16h
Hoechst
Annexin V
sh-ctrl
sh-USP21
sh-p53
sh-USP21
+sh-p53
200μm
RKO
0h
8h
16h
Apoptosis(%)
sh-ctrl
sh-USP21
sh-p53
sh-USP21
+sh-p53
sh-USP21+sh-p53
(h)
PARP
CL-PARP
p53
USP21
GAPDH
Pro-Cas-3
CL-Cas-3
RKO
H
Dox
0h
8h
16h
Hoechst
Annexin V
ctrl
F-USP21
F-USP21
+sh-Mdm2
sh-Mdm2
200μm
RKO
0h
8h
16h
Apoptosis(%)
F-USP21+
sh-Mdm2
(h)
PARP
CL-PARP
Mdm2
p53
USP21
GAPDH
Pro-Cas-3
CL-Cas-3
RKO
