# Supplementary figures and images for: USP21 functions as an oncogenic regulator of the Mdm2-p53 axis in colorectal cancer
Source: Cell Death Discov. 2026 May 22;12:305. doi: 10.1038/s41420-026-03170-3 (PMC13370000; doi:10.1038/s41420-026-03170-3)

Figure 1

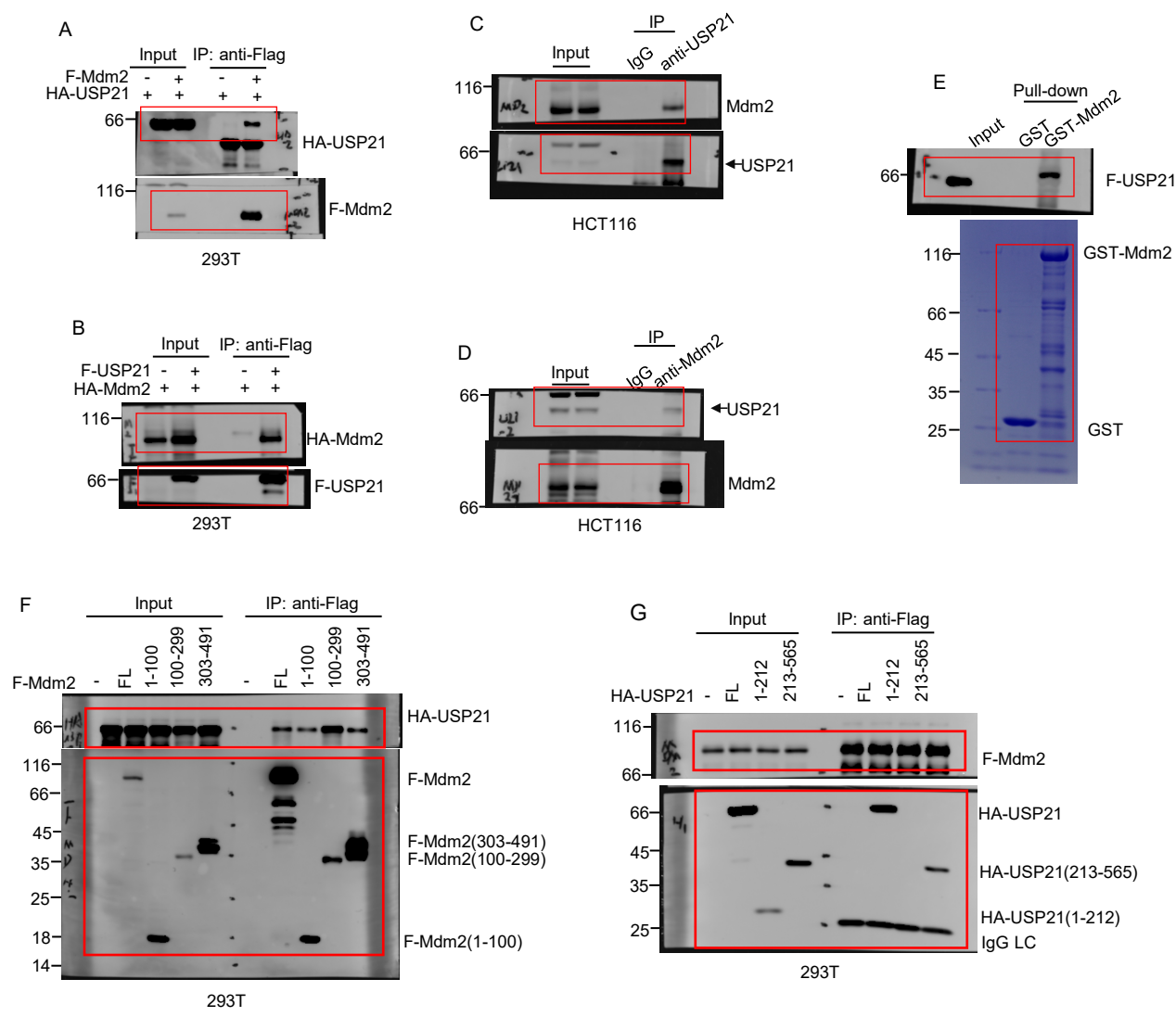

Figure 2

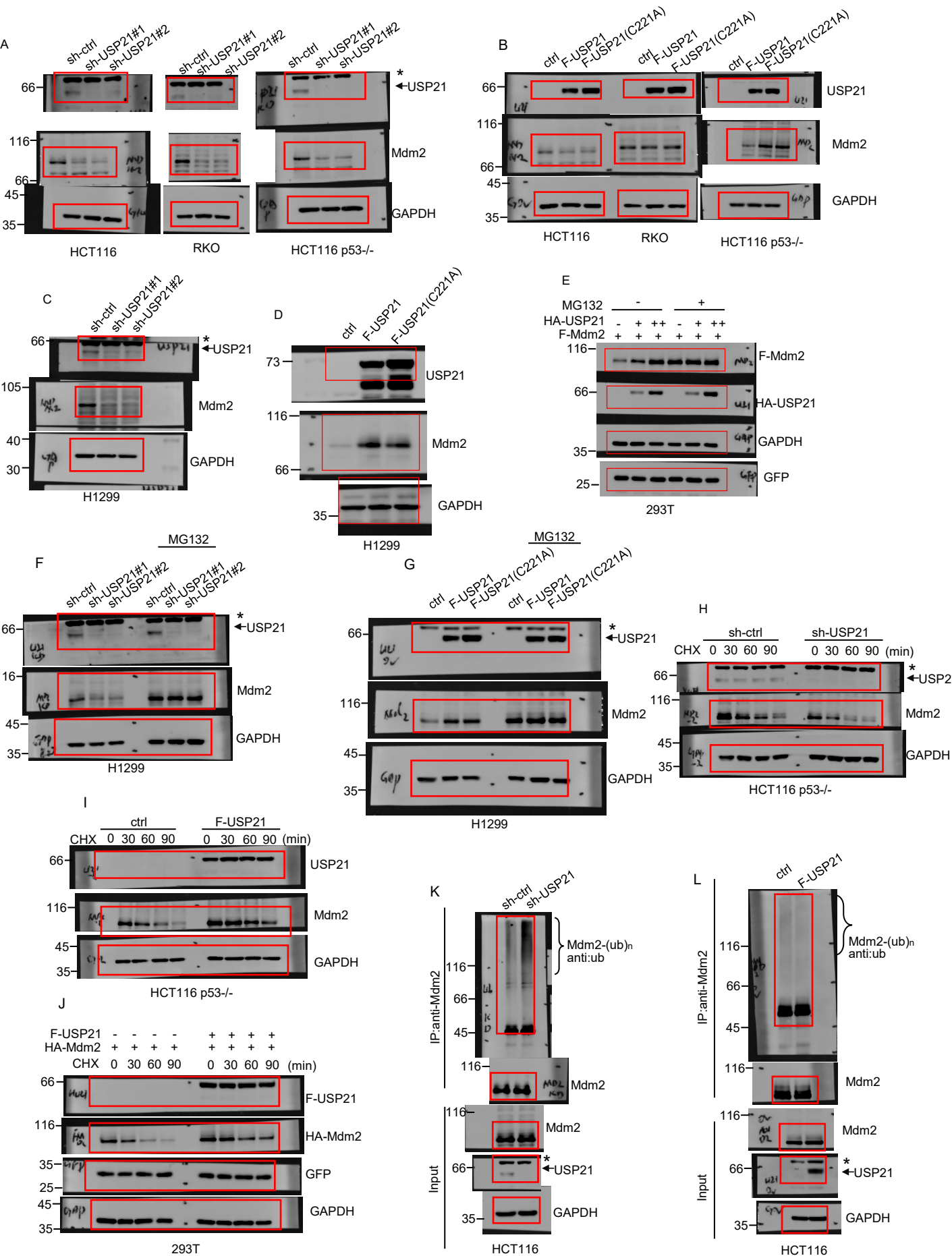

# Figure 3

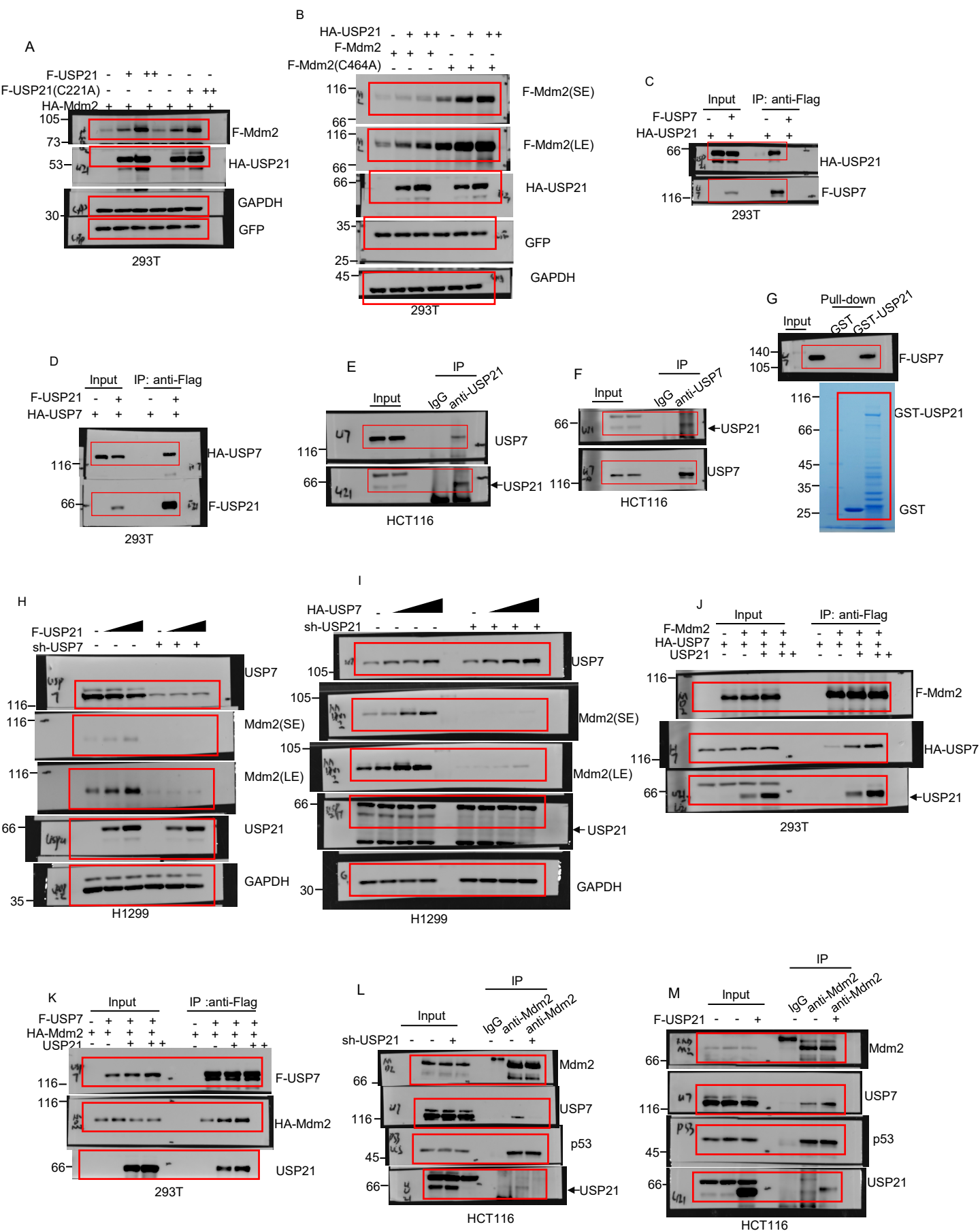

Figure 3

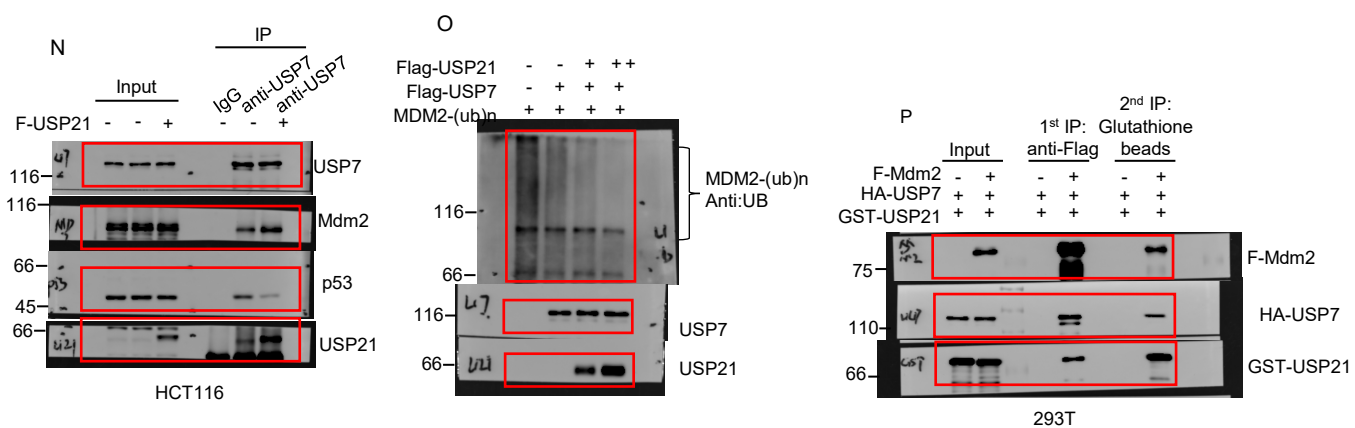

# Figure 4

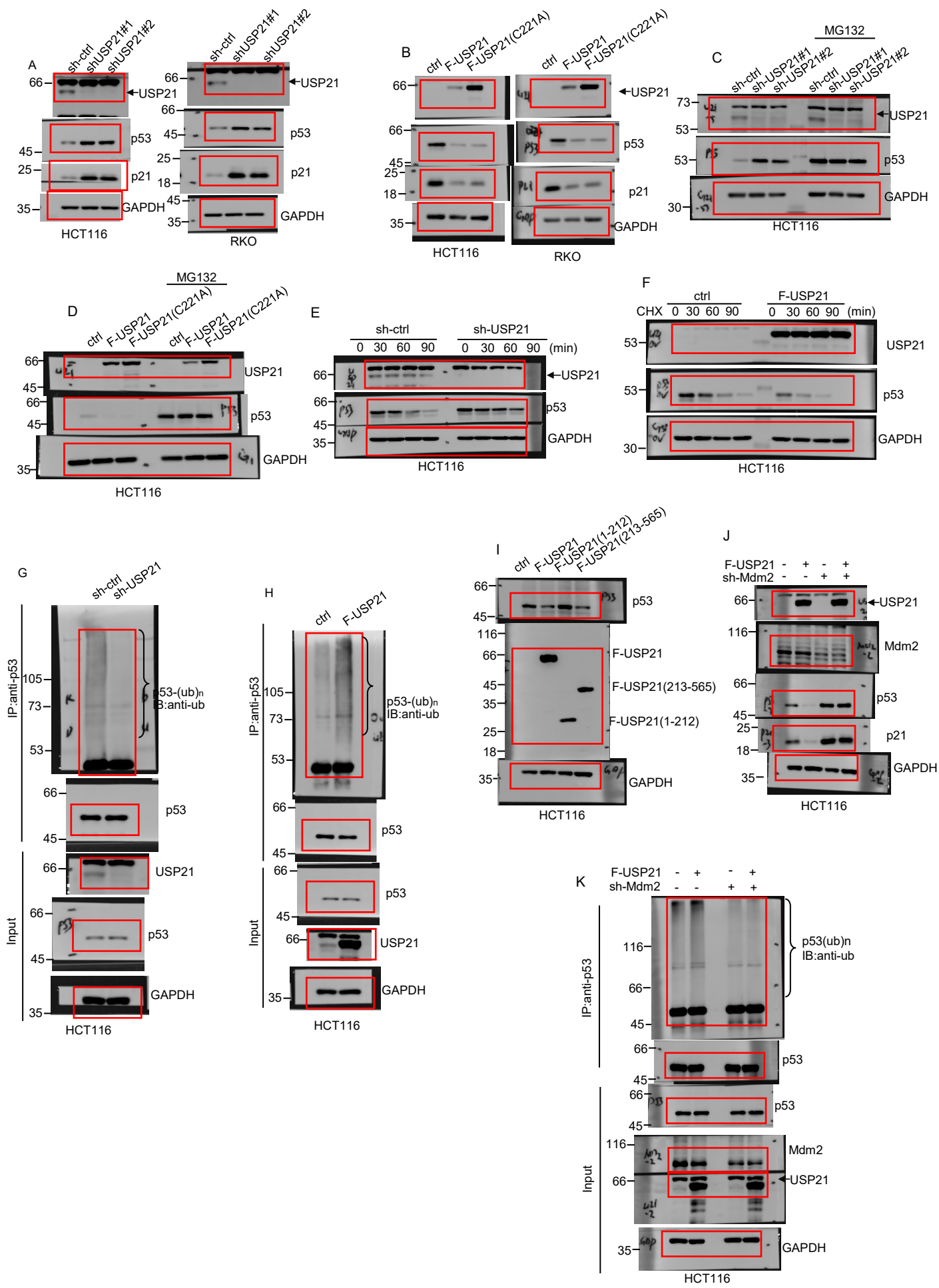

Figure 4

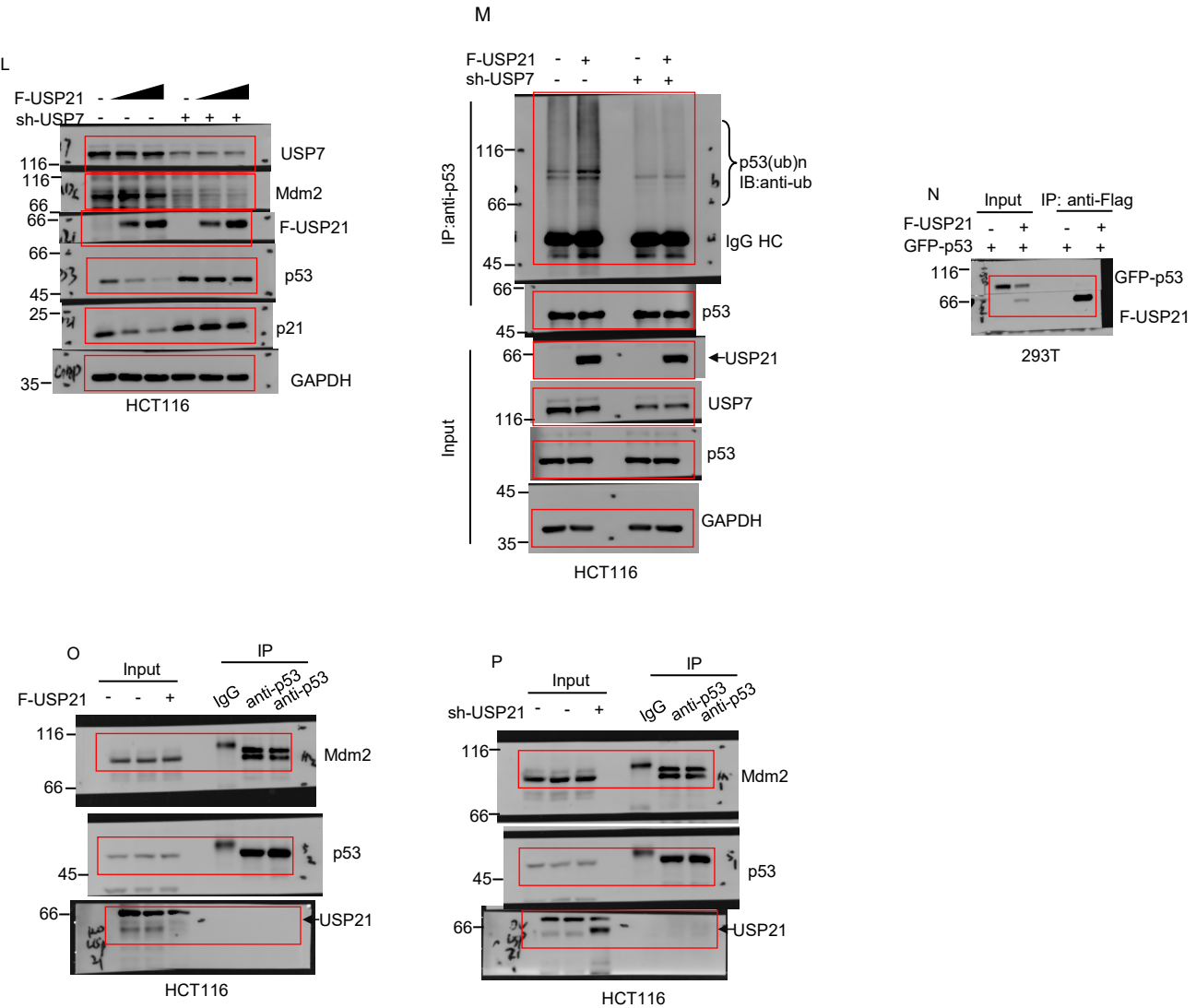

Figure 5

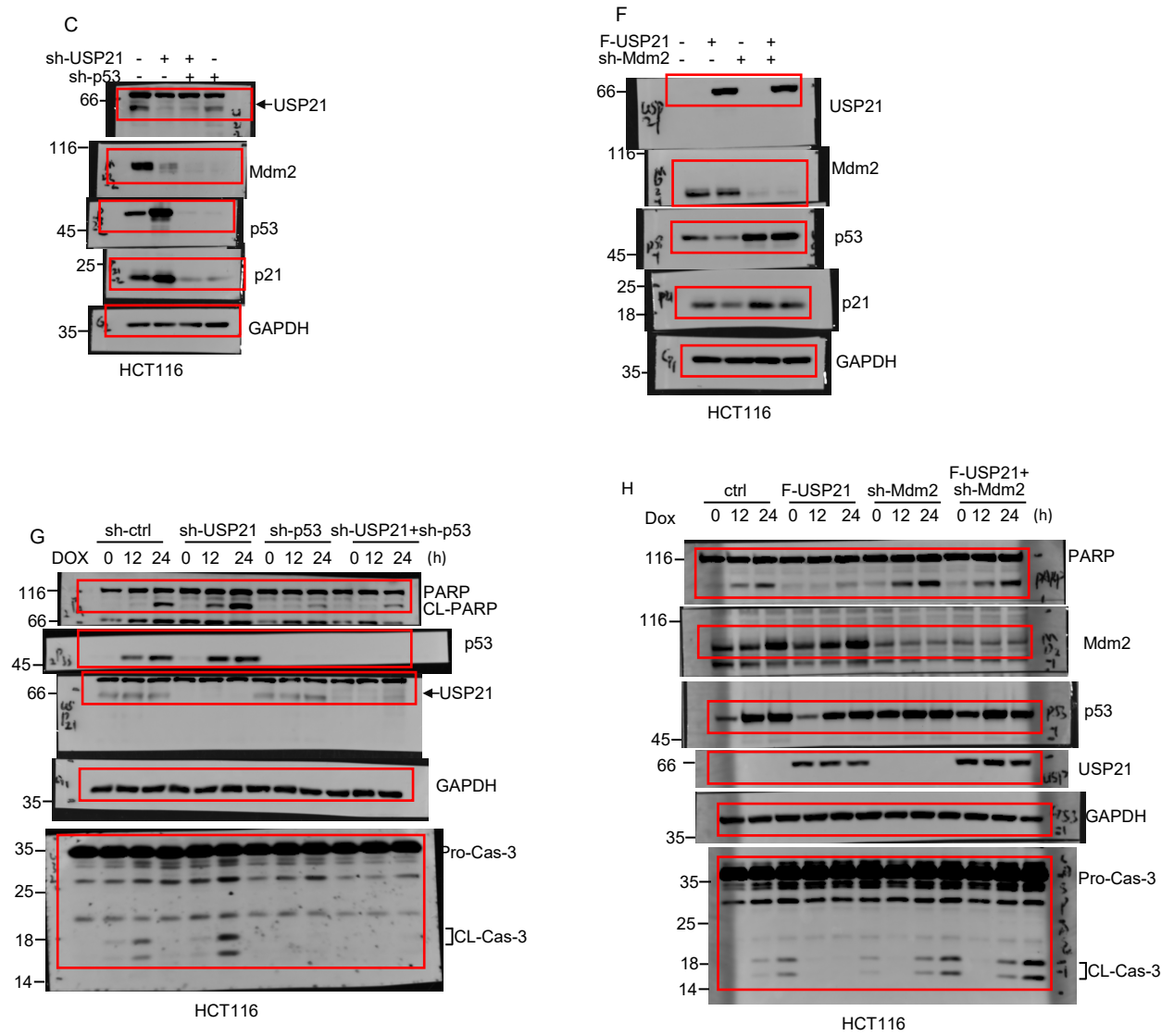

Figure 6

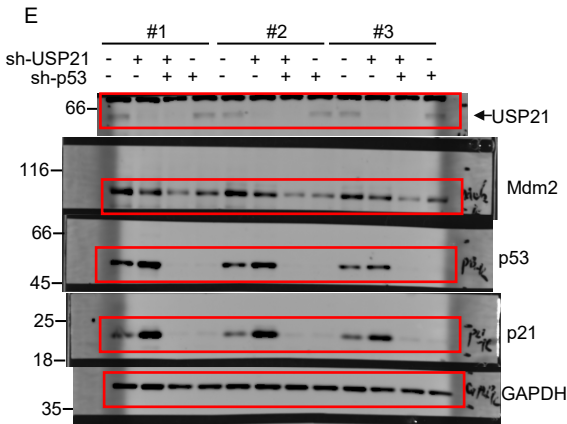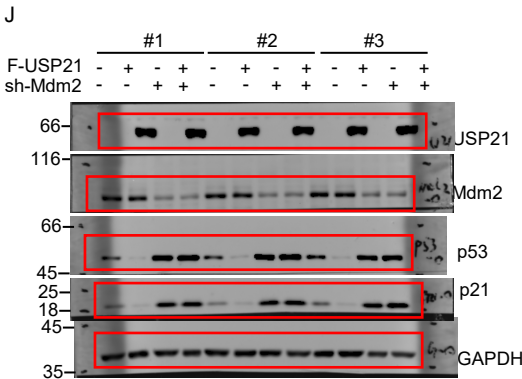

# Figure S1

A

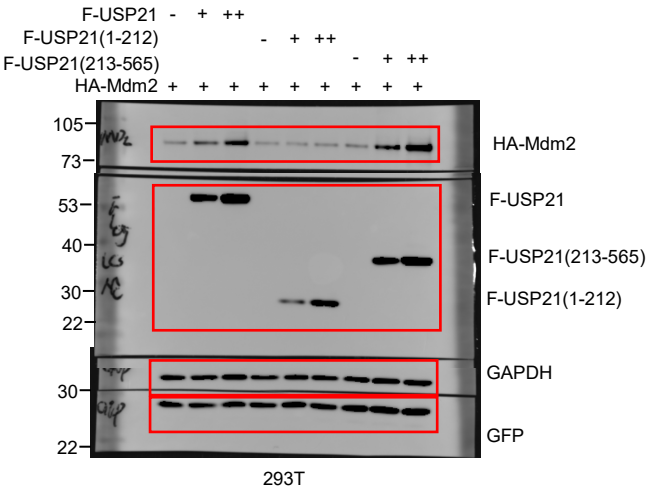

Figure S2

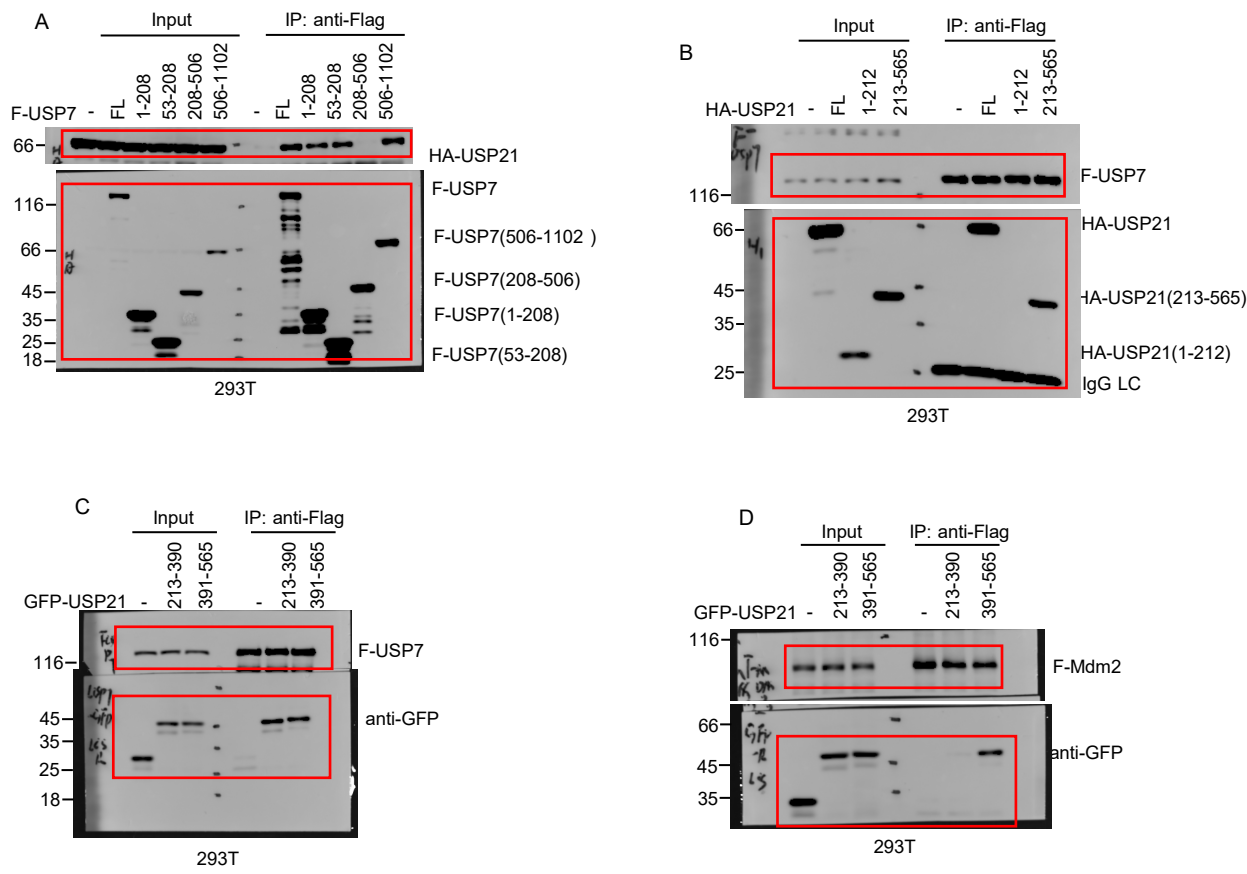

Figure S3

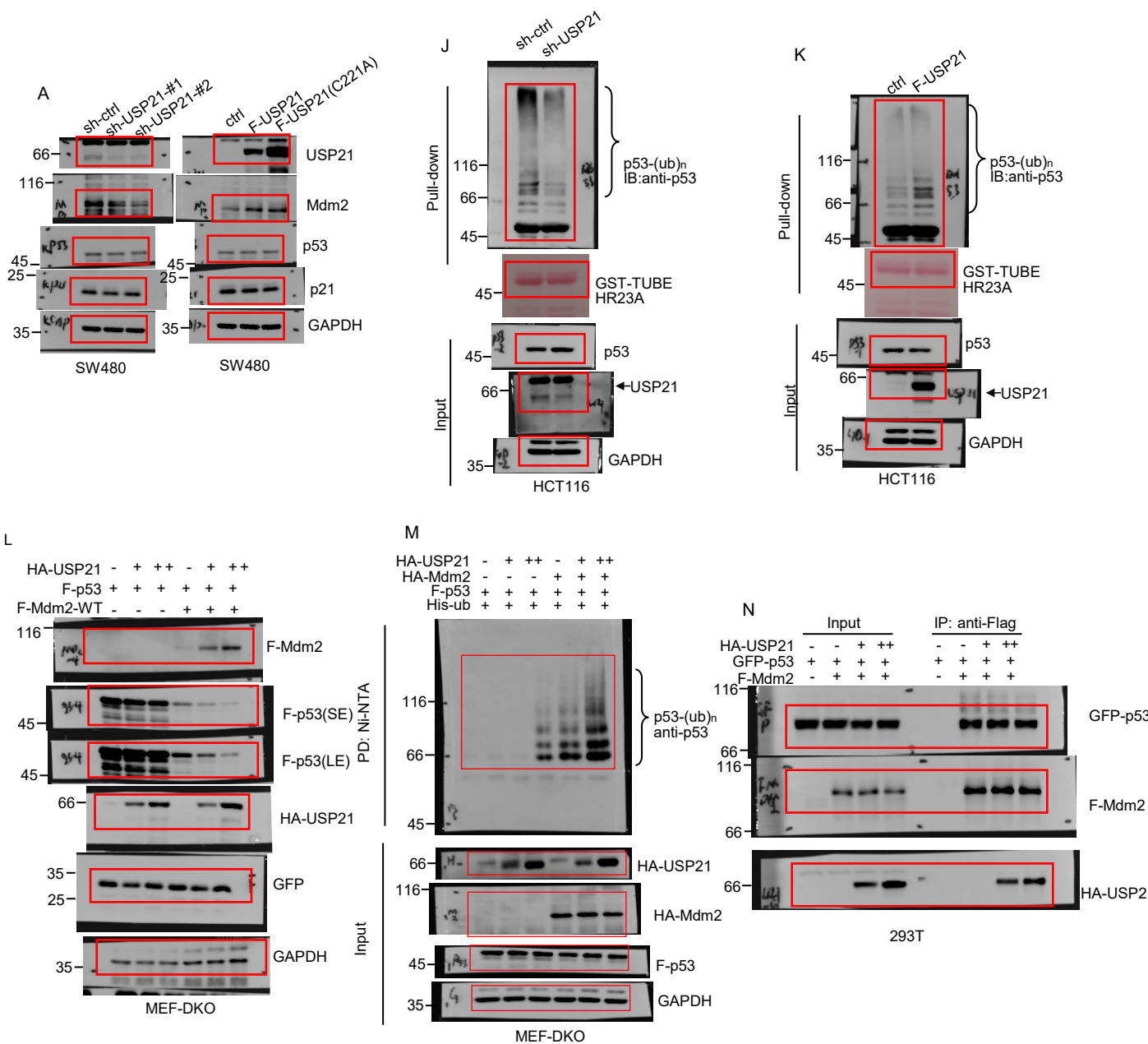

# Figure S4

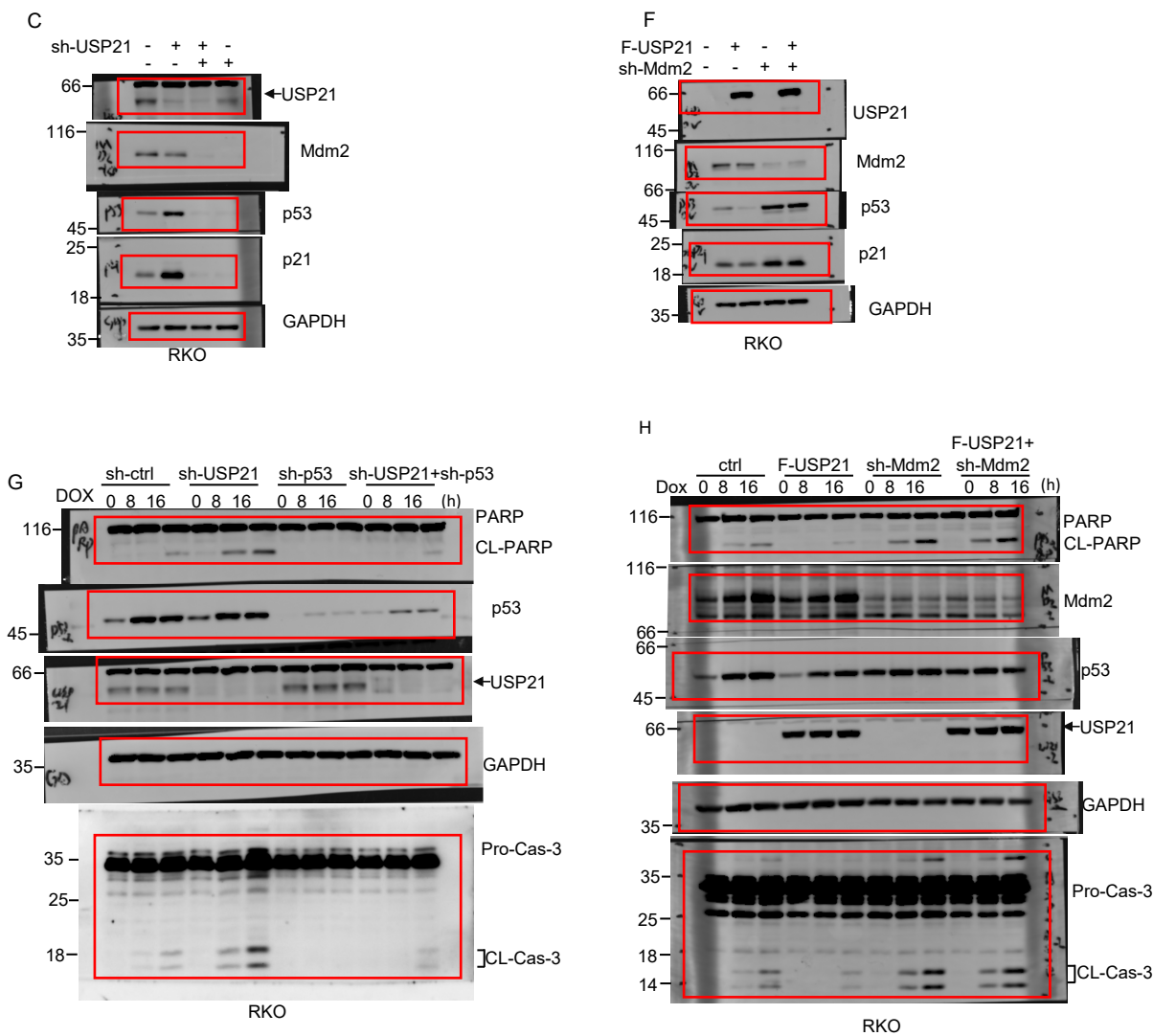

Supplement: Supplementary file 4 — Original data for western blot [file 41420_2026_3170_MOESM4_ESM.pdf]
